# Relationships between childhood adversity and inflammatory biomarkers in adulthood: A cross-sectional analysis of a middle-to older-aged population

**DOI:** 10.1016/j.ssmph.2024.101608

**Published:** 2024-01-12

**Authors:** Caroline Pitts, Seán R. Millar, Ivan J. Perry, Catherine M. Phillips

**Affiliations:** a School of Public Health, Physiotherapy and Sports Science, University College Dublin, Dublin 4, Ireland; b School of Public Health, University College Cork, Cork, Ireland

**Keywords:** Adverse childhood experiences, Inflammatory biomarkers, Cardiovascular disease, Household dysfunction

## Abstract

**Background:**

Exposure to adverse childhood experiences (ACEs) has been linked with increased cardiometabolic risk in adulthood. Low-grade systemic inflammation may underlie this association. Thus far, however, there has been limited investigation of later life inflammatory biomarkers in the context of childhood adversity.

**Objectives:**

To assess ACE history, and ACE subcategory, relationships with a broad range of inflammatory biomarkers in middle-to older-aged adults to test the hypothesis that ACE exposure is associated with an unfavourable inflammatory profile in adulthood and determine whether associations vary by ACE subtype and sex.

**Methods:**

This study used data from a random sample of 1,839 men and women aged 46–74 years. Participant exposure to ACEs (overall and subtypes including abuse, neglect and household dysfunction) was determined using a validated 10-item ACE questionnaire. Inflammatory biomarkers (pro-inflammatory cytokines, adipocytokines, acute-phase response proteins, white blood cell counts and their constituents, coagulation factors and glycoprotein acetyl) were measured from participant blood samples. Linear regression analyses examined relationships between ACE history (overall and each subcategory) and inflammatory biomarkers in adulthood, controlling for potential confounders. Sex-stratified and mediation analyses were also conducted.

**Results:**

In age and sex-adjusted models, ACE history was significantly associated with higher c-reactive protein (*p* = 0.027), resistin (*p* = 0.024), white blood cell count (WBC) (*p* = 0.034), monocyte (*p* = 0.044), eosinophil (*p* = 0.031) and plasminogen activator inhibitor-1 (*p* = 0.047) concentrations, and lower adiponectin (*p* = 0.025) levels. Results from stratified analyses indicated sex differences and ACE subtype specific associations, with household dysfunction identified as the main driver of positive ACE associations with WBCs and constituents (all *p* < 0.05). Mediation analyses suggested that BMI and smoking mediate relationships between ACE exposures and increased inflammation.

**Conclusions:**

This study provides evidence that ACE exposure may be associated with more pro-inflammatory and pro-thrombotic profiles in adulthood. Associations differed according to ACE subtype, and sex differences exist, which may influence cardiometabolic risk.

## Highlights

• Adverse childhood experiences are linked with later life cardiovascular disease.
• ACEs associated with more pro-inflammatory and pro-thrombotic profiles in adulthood.
• Sex differences and ACE subtype specific associations were observed.
• Household dysfunction main driver of associations with WBC counts and constituents.
• BMI and smoking mediate relationships between ACE exposure and inflammation.

## 1 Introduction

Events during the first 18 years of life are critical determinants of health outcomes in adulthood. Adverse childhood experiences (ACEs), including abuse, neglect and household dysfunction are prevalent, with up to 57.8% of adults in the United States reporting exposure to at least one ACE (Giano et al., 2020). Individuals exposed to ACEs have been found to have higher rates of morbidity and mortality in later life (Campbell et al., 2015), making childhood adversity an important area of public health research. Consequently, there is a growing body of research examining the relationships between ACEs and a range of later life health outcomes including chronic stress, substance abuse, mental illness and cardiovascular disease (CVD) (Petruccelli et al., 2019). Among these health outcomes, CVD is a major public health concern as it is a leading, but preventable, cause of death and contributor to disability worldwide (World Health Organization, 2019).

A life-course perspective explains the relationship between early life experiences and health outcomes in adulthood through three theories: critical period, accumulation of risk and the pathway model (Ben-Shlomo & Kuh, 2002). The critical period or ‘latency’ model proposes that exposure during a specific period of life, such as during development, has irreversible and lasting effects on body systems (Ben-Shlomo & Kuh, 2002) and biological mechanisms underlying the relationship between ACEs and CVD have been proposed whereby physiological disruption of regulatory systems by ACEs lead to altered metabolic, immune and neuroendocrine function (Suglia et al., 2017). The accumulation of risk model suggests that exposure to multiple risk factors contributes to disease risk across the life-course, and the level of exposure relates to the intensity of the outcome (Cable, 2014). The pathway model proposes that early life events are related to adult health outcomes through a series of ‘intervening risks’ (Cable, 2014). These theories are not mutually exclusive, and a combination of the models can be applied to conceptualise the relationship between ACEs and CVD. Exposure to extreme stress during critical periods of childhood may both disrupt immune system function and increase the likelihood of exposure to a range of adverse factors in later childhood and adult life (e.g. obesity, alcohol, tobacco and substance misuse, and low socioeconomic status), thereby contributing to increased inflammation throughout the life-course.

CVD risk factors have been studied in relation to ACE exposure (Baldwin & Danese, 2019; O'Leary et al., 2023; Scott et al., 2021; Zhu et al., 2022), revealing higher risk of obesity, diabetes and unfavourable lipid and inflammatory profiles in adulthood among individuals exposed to one or more ACE compared to non-exposed individuals (Baldwin & Danese, 2019; O'Leary et al., 2023; Zhu et al., 2022). Thus far, research on ACEs and inflammation has focused mainly on c-reactive protein (CRP), tumour necrosis factor alpha (TNF-α) and interleukin 6 (IL-6) (Carpenter et al., 2010; Carroll et al., 2013; Hostinar et al., 2015; Iob et al., 2020; Kuzminskaite et al., 2020; Smith et al., 2011). Of the ACE subtypes, abuse has been most frequently associated with increased concentrations of CRP, TNF-a and IL-6 (Bertone-Johnson et al., 2012; Kiecolt-Glaser et al., 2011; Kraynak et al., 2019; Lacey et al., 2020; Pereira et al., 2019; Smith et al., 2011). Sex differences in ACE exposure have also been identified, with females being more likely than males to report exposure to sexual abuse, physical neglect, emotional neglect, alcohol and/or drug abuse in the household, and a household member with a serious mental illness (Haahr-Pedersen et al., 2020).

In the context of ACEs, investigation of biomarkers representing different aspects of cellular and organ sources of inflammation (including anti-inflammatory biomarkers) in adulthood is lacking. This is especially important considering the complex inter-relationships between certain inflammatory biomarkers (Fruhbeck et al., 2018; Tanaka et al., 2014). Furthermore, examination of sex-specific ACE associations with inflammatory biomarkers in later life has been relatively under-researched. We address these research gaps by investigating ACE history and ACE subcategory associations with a broad range of inflammatory biomarkers, including pro-inflammatory cytokines and adipocytokines, acute-phase response proteins, white blood cell counts (WBC) and their constituents (measures of chronic inflammation), coagulation factors and novel glycoprotein acetyl (GlycA), which represents the concentration and glycosylation of acute phase proteins released during states of inflammation (Chiesa et al., 2022), in order to provide a comprehensive view of inflammation in the body. Using a random sample of 1,839 middle-to older-aged Irish men and women, we test the hypothesis that ACE history is associated with unfavourable inflammatory profiles in adulthood and examine whether associations vary by ACE subtype and sex.

## 2 Materials and methods

### 2.1 Study population

The Cork and Kerry Diabetes and Heart Disease Study (Phase II – Mitchelstown cohort) was a cross-sectional study which recruited a sample of middle-to older-aged men and women living in the Mitchelstown area of County Cork, Ireland between 2010 and 2011. Full details of the study, which aimed to examine major CVD risk factors, have been described previously (Kearney et al., 2013). In brief, participants were recruited through a primary care centre, the Living Health Clinic, using stratified random sampling. Of the initial 3,807 individuals invited to participate, 2,047 middle-to older-aged adults (49% male; age range: 46–74 years) completed the baseline assessment, including a questionnaire and physical examination (67% response rate). Ethics committee approval conforming to the Declaration of Helsinki was granted from the Clinical Research Ethics Committee of University College Cork. All participants provided signed informed consent for their data to be used for research purposes. After the exclusion of participants with missing or incomplete ACE data (n = 208), the current analysis is based on 1,839 participants.

### 2.2 Adverse childhood experiences

Data on ACE exposures were collected using a 10-item ACE questionnaire as previously described (O'Leary et al., 2023). The questionnaire is a validated instrument including questions on abuse (emotional, physical and sexual), neglect (emotional and physical) and household dysfunction (parental separation/divorce, domestic violence, substance abuse, mental illness and incarceration of a family member) (Anda et al., 2010). All questions, which refer to a participant's first 18 years of life, were answered with a binary response (yes/no). Total ACE scores were calculated and ranged from 0 to 8 as no participants reported exposure to all 10 ACE items. A binary ACE variable based on history of ACEs (yes/no) was then generated. ‘Yes’ responses were further classified according to ACE subtype.

### 2.3 Clinical procedures and biomarker profiling

Study participants attended the clinic in the morning after an overnight fast and blood samples were taken on arrival. Fasting glucose and glycated haemoglobin A_1c_ (HbA_1c_) concentrations were measured in fresh samples by Cork University Hospital Biochemistry Laboratory using standardised procedures. Glucose concentrations were determined using a glucose hexokinase assay (Olympus Life and Material Science Europa Ltd., Lismeehan, Co. Clare, Ireland) and HbA_1c_ levels were measured in the haematology laboratory on an automated high-pressure liquid chromatography instrument Tosoh G7 [Tosoh HLC-723 (G7), Tosoh Europe N.V, Tessenderlo, Belgium].

Inflammatory biomarker profiling has been previously described (Phillips et al., 2017). In brief, a biochip array system (Evidence Investigator; Randox Laboratories, Antrim, UK) analysed CRP, TNF-α, IL-6, adiponectin, leptin, resistin and plasminogen activator inhibitor-1 (PAI-1) concentrations. An immunoturbidimetric assay (Rx. Daytona; Randox Laboratories, Antrim, UK) determined complement component 3 (C3) concentrations. Inter- and intra-assay coefficients of variation were <10% for the biochip array and <5% for the immunoturbidimetric assay (Randox Biosciences, 2023). WBCs and WBC constituents (monocytes, basophils, eosinophils, neutrophils and lymphocytes) were determined using flow cytometry technology in the Cork University Hospital Haematology Laboratory. Serum glycoprotein A (glycA) was measured on serum specimens using nuclear magnetic resonance spectroscopy (*NMR LipoProfile*® analysis) at LipoScience Inc (Raleigh, NC, USA) (Otvos et al., 2015). The leptin-adiponectin ratio (LAR) and neutrophil-lymphocyte ratio (NLR) were calculated.

### 2.4 Covariates

Anthropometric measurements were performed by trained researchers with reference to a standard operating procedures manual. Height was measured with a portable Seca Leicester height/length stadiometer (Seca, Birmingham, UK) and weight was measured using a portable electronic Tanita WB-100MA weighing scale (Tanita Corp, IL, USA). The weighing scale was placed on a firm flat surface and was calibrated weekly. Body mass index (BMI = weight (kg)/height(m)^2^) was calculated from measured weight and height.

Diet was evaluated using a modified version of the self-completed European Prospective Investigation into Cancer and Nutrition (EPIC) Food Frequency Questionnaire (FFQ) (Riboli et al., 1997), which has been validated extensively in several populations (Bingham et al., 1997). Adapted to reflect the Irish diet, the 150-item semi-quantitative FFQ used in the current study was originally validated for use in the Irish population using food diaries and a protein biomarker in a volunteer sample (Harrington, 1997) and incorporated into the SLÁN Irish National Surveys of Lifestyle Attitudes and Nutrition 1998, 2002; 2007; Friel et al., 1997; Kelleher et al., 2003; Morgan et al., 2008). The average medium serving of each food item consumed by participants over the last 12 months was converted into quantities using standard portion sizes. Food item quantity was expressed as (g/d) and beverages as (ml/d). The daily intake of energy and nutrients was computed from FFQ data using a tailored computer programme (FFQ Software Version 1.0; developed by the National Nutrition Surveillance Centre, School of Public Health, Physiotherapy and Sports Science, University College Dublin, Belfield, Dublin 4, Ireland), which linked frequency selections with the food equivalents in McCance and Widdowson Food Tables (Sokol et al., 2016).

Based on the FFQ, the Dietary Approaches to Stop Hypertension (DASH) score was constructed to assess diet quality. DASH is a dietary pattern rich in fruits, vegetables, whole grains and low-fat dairy foods and is limited in sugar-sweetened foods and beverages, red meat and added fats. This diet has been promoted by the National Heart, Lung and Blood Institute (part of the National Institutes of Health, a United States government organisation) to prevent and control hypertension. DASH diet scores ranged from 11 to 42. Lower scores represent poorer and higher scores represent better quality diet (Harrington et al., 2013).

Participants completed a general health questionnaire which included questions on age and sex, education, use of anti-inflammatory medications, morbidity and lifestyle behaviours. Categories of education included ‘some primary (not complete)’, ‘primary or equivalent’, ‘intermediate/group certificate or equivalent’, ‘leaving certificate or equivalent’, ‘diploma/certificate’, ‘primary university degree’ and ‘postgraduate/higher degree’. These were collapsed into a binary variable: ‘primary education only’ (finished full-time education at age 13 years or younger) and ‘intermediate or higher’. Type 2 diabetes was determined as a fasting glucose level ≥7.0 mmoL/l or HbA_1c_ level ≥6.5% (≥48 mmol/mol) (American Diabetes Association, 2014) or by self-reported diagnosis. The presence of CVD was obtained by asking study participants if they had been diagnosed with any one of the following seven conditions: Heart Attack (including coronary thrombosis or myocardial infarction), Heart Failure, Angina, Aortic Aneurysm, Hardening of the Arteries, Stroke or any other Heart Trouble. Subjects who indicated a diagnosis of any one of these conditions were classified as having CVD. Smoking status was defined as follows: ‘never smoked’, i.e. having never smoked at least 100 cigarettes (5 packs) in their entire life; ‘former smoker’, i.e. having smoked 100 cigarettes in their entire life and do not smoke at present; and ‘current smoker’, i.e. smoking at present. Alcohol use was categorised as ‘never’ (<1 standard drink a week), ‘moderate” (between 1 and 14 standard drinks a week), and ‘heavy’ (>14 standard drinks a week).

### 2.5 Statistical analysis

Descriptive characteristics for the full sample, and according to sex and ACE history, were examined. Categorical features are presented as percentages and continuous variables are shown as a mean plus or minus one standard deviation (SD) or as a median and interquartile range (IQR) for skewed data. Differences between groups based on sex and ACE exposure were analysed using a Pearson's chi-square test, Student's *t*-test or a Mann Whitney *U* test. Skewed biomarker data were log-transformed and linear regression analyses were used to examined ACE history and ACE subcategory associations with inflammatory biomarkers, overall and stratified by sex. Four regression models were run; Model 1 was a crude unadjusted model, Model 2 was adjusted for age and sex (entire sample only) and Model 3 was additionally adjusted for anti-inflammatory medication use, type 2 diabetes, CVD history and cancer. A fourth model also adjusted for lifestyle behaviours (smoking status, alcohol use, diet quality) and BMI. In fully adjusted models, to correct for multiple comparisons, we calculated false discovery rate (FDR) adjusted *p* values (included as Supplementary Information) via the Romano-Wolf multiple hypothesis correction method using the **rwolf** command in Stata (Clarke et al., 2019). Data analyses were conducted using Stata SE Version 13 (Stata Corporation, College Station, TX, USA) for Windows. For all analyses, a *p* value (two-tailed) of less than 0.05 was considered to indicate statistical significance.

To further explore whether relationships between ACEs and inflammatory biomarkers are mediated by lifestyle factors and BMI, we conducted mediation analyses. For any biomarker that demonstrated a significant relationship with any ACE history or ACE subcategory in Model 3 for the full sample, we calculated direct and indirect effects with 95% confidence intervals determined from 5000 bootstrap samples using the **PROCESS** macro (Hayes, 2017) in IBM SPSS Statistics 25 (IBM Corp., Armonk, NY, USA). We also performed the Sobel test of mediation using the **sgmediation2** command in Stata (Mize, 2023). Evidence of mediation was considered on the basis of an indirect effect with confidence intervals that did not include the null value and/or a Sobel test *p* value less than 0.05.

## 3 Results

### 3.1 Descriptive characteristics

Supplementary Table 1 presents the total number and percentage of participants, for the entire sample and stratified by sex, who reported exposure to any ACE and ACE subcategory. The analytic sample consisted of 1,839 subjects (49% male; age range: 46–74 years; median age: 59.5). Overall, 22.6% of participants reported exposure to at least one ACE. Within the ACE subcategories, household dysfunction was the most frequently reported ACE (14.7%), followed by abuse (12.2%). There were no significant sex differences regarding ACE exposure, overall or by subtype. Supplementary Table 2 further breaks down the ACE questionnaire and presents the total number and percentage of responses to each ACE item for the entire sample and stratified by sex.

Participant characteristics and inflammatory profiles for the entire sample, and stratified by sex and ACE history, are presented in Table 1. A higher percentage of male participants were educated to a primary level only, were using anti-inflammatory medications and were former smokers and heavy drinkers (*p* < 0.001). Male subjects also had poorer diet quality as indicated by lower DASH scores and had higher mean BMI (*p* < 0.001). Males were additionally more likely to have type 2 diabetes and a history of CVD, while females were more likely to report a diagnosis of cancer. With regard to ACE history, participants who indicated any ACE exposure were more likely to report a past history of CVD (*p* = 0.017); a higher percentage also indicated having been a former smoker (*p* = 0.033) and moderate or heavy alcohol use (*p* = 0.017). Higher mean BMI levels were also observed among subjects reporting any ACE history compared to study participants who did not indicate an ACE exposure (*p* = 0.025).

**Table 1:** Participant characteristics and inflammatory profiles for the full sample and according to sex and any ACE history.

Characteristic	Level	All	Sex		*p*	Any ACE history		*p*
		(n = 1839)	Male (n = 904)	Female (n = 935)		Yes (n = 416)	No (n = 1423)	
Age (years)	median (IQR)	59.5 (55.0, 64.0)	59.4 (55.0, 64.1)	59.6 (55.0, 64.0)	0.752	57.4 (53.6, 62.3)	60.5 (55.3, 64.5)	0.2
Primary education only	n (%)	467 (25.4)	266 (29.4)	201 (21.5)	< **0.001**	100 (24.0)	367 (25.8)	0.47
Anti-inflammatory medication use	n (%)	364 (19.8)	209 (23.1)	155 (16.6)	< **0.001**	76 (18.3)	288 (20.2)	0.375
Type 2 diabetes	n (%)	160 (8.7)	98 (10.9)	62 (6.6)	**0.001**	37 (8.9)	123 (8.6)	0.876
Cardiovascular disease	n (%)	194 (10.5)	130 (14.4)	64 (6.8)	< **0.001**	57 (13.7)	137 (9.6)	**0.017**
Cancer	n (%)	73 (4.0)	22 (2.4)	51 (5.5)	**0.001**	17 (4.1)	56 (3.9)	0.89
Never smoked	n (%)	928 (51.8)	380 (43.3)	548 (59.9)	< **0.001**	189 (46.4)	739 (53.3)	**0.033**
Former smoker	n (%)	610 (34.0)	371 (42.3)	239 (26.1)		159 (39.1)	451 (32.5)	
Current smoker	n (%)	255 (14.2)	127 (14.5)	128 (14.0)		59 (14.5)	196 (14.1)	
Non-drinker	n (%)	880 (47.9)	353 (39.0)	527 (56.4)	< **0.001**	175 (42.1)	705 (49.5)	**0.017**
Moderate drinker	n (%)	783 (42.6)	392 (43.4)	391 (41.8)		192 (46.2)	591 (41.5)	
Heavy drinker	n (%)	176 (9.6)	159 (17.6)	17 (1.8)		49 (11.8)	127 (8.9)	
Diet quality (DASH score)	mean (SD)	26.8 ± 5.4	25.0 ± 5.1	28.6 ± 5.1	< **0.001**	26.7 ± 5.6	26.9 ± 5.4	0.511
BMI (kg/m^2^)	mean (SD)	28.6 ± 4.7	29.2 ± 4.1	28.0 ± 5.1	< **0.001**	29.0 ± 4.8	28.5 ± 4.6	**0.025**
CRP (mg/L)	median (IQR)	1.34 (0.97, 2.26)	1.31 (0.95, 2.11)	1.37 (0.98, 2.39)	0.059	1.39 (0.96, 2.44)	1.32 (0.97, 2.22)	0.328
C3 (mg/dL)	mean (SD)	136.0 ± 24.3	134.3 ± 21.6	137.7 ± 26.5	**0.002**	136.5 ± 25.6	135.9 ± 23.9	0.642
IL-6 (pg/mL)	median (IQR)	1.77 (1.19, 2.85)	1.90 (1.27, 3.04)	1.66 (1.12, 2.69)	< **0.001**	1.84 (1.18, 2.86)	1.75 (1.20, 2.86)	0.927
TNF-α (pg/mL)	median (IQR)	5.95 (4.88, 7.27)	6.00 (5.00, 7.38)	5.90 (4.76, 7.15)	**0.008**	5.94 (4.88, 7.22)	5.95 (4.88, 7.29)	0.874
Leptin (ng/mL)	median (IQR)	1.95 (1.09, 3.15)	1.59 (0.87, 2.59)	2.25 (1.27, 4.21)	< **0.001**	1.82 (1.05, 2.97)	2.00 (1.10, 3.21)	0.236
Adiponectin (ug/mL)	median (IQR)	4.73 (2.91, 7.44)	3.26 (2.21, 4.92)	6.63 (4.43, 9.60)	< **0.001**	4.42 (2.82, 6.46)	4.86 (2.95, 7.69)	**0.001**
LAR	median (IQR)	0.42 (0.18, 0.85)	0.47 (0.21, 0.87)	0.36 (0.15, 0.81)	< **0.001**	0.43 (0.20, 0.85)	0.40 (0.18, 0.85)	0.256
Resistin (ng/mL)	median (IQR)	5.06 (3.92, 6.74)	4.88 (3.79, 6.52)	5.25 (4.00, 7.01)	**0.002**	5.20 (4.05, 6.99)	4.99 (3.88, 6.67)	0.068
GlycA (mmol/L)	mean (SD)	409.0 ± 63.6	394.7 ± 62.8	422.8 ± 61.3	< **0.001**	410.5 ± 69.9	408.6 ± 61.7	0.591
WBC (10^9^/L)	median (IQR)	5.70 (4.80, 6.80)	5.90 (5.00, 7.00)	5.50 (4.60, 6.50)	< **0.001**	5.90 (4.90, 7.10)	5.70 (4.80, 6.70)	**0.041**
Monocytes (10^9^/L)	median (IQR)	0.50 (0.41, 0.62)	0.54 (0.44, 0.68)	0.46 (0.37, 0.57)	< **0.001**	0.51 (0.42, 0.64)	0.50 (0.40, 0.61)	0.111
Basophils (10^9^/L)	median (IQR)	0.032 (0.02, 0.04)	0.032 (0.02, 0.04)	0.032 (0.02, 0.04)	0.644	0.033 (0.02, 0.04)	0.032 (0.02, 0.04)	0.067
Eosinophils (10^9^/L)	median (IQR)	0.17 (0.11, 0.26)	0.19 (0.12, 0.29)	0.16 (0.10, 0.23)	< **0.001**	0.18 (0.12, 0.27)	0.17 (0.11, 0.25)	0.056
Neutrophils (10^9^/L)	median (IQR)	3.12 (2.52, 3.92)	3.26 (2.63, 4.14)	2.99 (2.41, 3.76)	< **0.001**	3.21 (2.51, 4.08)	3.10 (2.52, 3.89)	0.235
Lymphocytes (10^9^/L)	median (IQR)	1.75 (1.43, 2.14)	1.73 (1.41, 2.13)	1.76 (1.44, 2.16)	0.273	1.79 (1.45, 2.21)	1.74 (1.42, 2.13)	0.063
NLR	median (IQR)	1.78 (1.40, 2.28)	1.86 (1.49, 2.37)	1.68 (1.31, 2.18)	< **0.001**	1.76 (1.37, 2.25)	1.79 (1.41, 2.29)	0.271
PAI-1 (ng/mL)	mean (SD)	27.2 ± 12.3	28.8 ± 12.4	25.7 ± 12.0	< **0.001**	28.3 ± 12.9	26.9 ± 12.1	**0.037**

Data are presented as mean (SD) or median (IQR) for continuous variables and number and (%) for categorical variables. *p* for difference determined from a chi-square test, an independent samples *t*-test or a Mann-Whitney *U* test. Significant *p* in **bold**.

C3: complement component 3; CRP: c-reactive protein; DASH: Dietary Approaches to Stop Hypertension; GlycA: glycoprotein acetyl; IL-6: interleukin 6; LAR: leptin-adiponectin ratio; NLR: neutrophil-lymphocyte ratio; PAI-1: plasminogen activator inhibitor-1; TNF-a: tumour necrosis factor alpha; WBC: white blood cell count.

Regarding inflammatory profiles, more pro-inflammatory levels of C3 (*p* = 0.002), leptin (*p* < 0.001), resistin (*p* = 0.002) and glycA (*p* < 0.001) were found in female participants. Among males, there were more pro-inflammatory levels of IL-6 (*p* < 0.001), TNF-α (*p* = 0.008), WBCs (*p* < 0.001), monocytes (*p* < 0.001), eosinophils (*p* < 0.001), neutrophils (*p* < 0.001), NLR (*p* = 0.001) and PAI-1 (*p* < 0.001) relative to females. Lower (more pro-inflammatory) adiponectin concentrations were also observed in males, resulting in a higher LAR (p < 0.001). Individuals with a history of exposure to any ACE had lower levels of adiponectin (*p* = 0.001) and higher levels of WBCs (*p* = 0.041) and PAI-1 (*p* = 0.037).

### 3.2 Descriptive statistics according to ACE subcategories

Table 2 presents descriptive characteristics and inflammatory profiles according to each ACE subcategory. With regard to lifestyle factors, significant differences in current or former tobacco use according to the abuse (*p* = 0.028) and household dysfunction (*p* = 0.011) subtypes were observed, with subjects reporting household dysfunction exposure also indicating heavier alcohol use compared to those who did not (*p* = 0.003). Mean BMI levels were also significantly higher among participants who reported having been exposed to abuse (*p* = 0.046) and neglect (*p* = 0.018), compared to those who did not report exposure to these ACE subtypes.

**Table 2:** Participant characteristics and inflammatory profiles according to ACE subcategories – full sample.

Characteristic	Abuse exposure		*p*	Neglect exposure		*p*	Household dysfunction exposure		*p*
	Yes (n = 225)	No (n = 1614)		Yes (n = 119)	No (n = 1720)		Yes (n = 271)	No (n = 1568)	
Age (years)	60.1 (55.3, 64.5)	57.4 (53.8, 62.3)	<**0.001**	60.0 (55.2, 64.3)	57.9 (53.8, 61.6)	<**0.001**	60.0 (55.2, 64.4)	57.0 (53.6, 61.5)	<**0.001**
Primary education only	59 (26.2)	408 (25.3)	0.761	35 (29.4)	432 (25.1)	0.298	60 (22.1)	407 (26.0)	0.183
Anti-inflammatory medication use	47 (20.9)	317 (19.6)	0.66	20 (16.8)	344 (20.0)	0.398	45 (16.6)	319 (20.3)	0.154
Type 2 diabetes	22 (9.8)	138 (8.6)	0.542	11 (9.2)	149 (8.7)	0.829	24 (8.9)	136 (8.7)	0.924
Cardiovascular disease	32 (14.2)	162 (10.0)	0.056	10 (8.4)	184 (10.7)	0.431	41 (15.1)	153 (9.8)	**0.008**
Cancer	8 (3.6)	65 (4.0)	0.734	7 (5.9)	66 (3.8)	0.325	11 (4.1)	62 (4.0)	0.935
Never smoked	95 (43.4)	833 (52.9)	**0.028**	57 (50.0)	871 (51.9)	0.643	115 (43.2)	813 (53.2)	**0.011**
Former smoker	89 (40.6)	521 (33.1)		43 (37.7)	567 (33.8)		106 (39.8)	504 (33.0)	
Current smoker	35 (16.0)	220 (14.0)		14 (12.3)	241 (14.4)		45 (16.9)	210 (13.8)	
Non-drinker	96 (42.7)	784 (48.6)	0.248	58 (48.7)	822 (47.8)	0.743	108 (39.9)	772 (49.2)	**0.003**
Moderate drinker	106 (47.1)	677 (41.9)		52 (43.7)	731 (42.5)		125 (46.1)	658 (42.0)	
Heavy drinker	23 (10.2)	153 (9.5)		9 (7.6)	167 (9.7)		38 (14.0)	138 (8.8)	
Diet quality (DASH score)	26.7 ± 5.7	26.8 ± 5.4	0.761	27.0 ± 5.5	26.8 ± 5.4	0.681	26.8 ± 5.5	26.8 ± 5.4	0.986
BMI (kg/m^2^)	29.2 ± 5.0	28.5 ± 4.6	**0.046**	29.8 ± 5.7	28.5 ± 4.6	**0.018**	29.0 ± 4.7	28.5 ± 4.7	0.088
CRP (mg/L)	1.36 (0.94, 2.07)	1.33 (0.97, 2.26)	0.805	1.42 (0.99, 2.68)	1.33 (0.97, 2.25)	0.290	1.41 (0.94, 2.72)	1.32 (0.97, 2.16)	0.158
C3 (mg/dL)	137.1 ± 24.7	135.9 ± 24.2	0.498	138.0 ± 28.6	135.9 ± 23.9	0.370	136.9 ± 26.8	135.9 ± 23.8	0.528
IL-6 (pg/mL)	1.86 (1.20, 2.91)	1.75 (1.19, 2.85)	0.538	1.95 (1.23, 3.46)	1.75 (1.19, 2.85)	0.200	1.86 (1.18, 2.99)	1.75 (1.20, 2.83)	0.358
TNF-a (pg/mL)	5.99 (4.87, 7.23)	5.94 (4.88, 7.27)	0.653	6.10 (4.74, 7.22)	5.94 (4.89, 7.28)	0.645	5.94 (4.91, 7.16)	5.95 (4.87, 7.28)	0.831
Leptin (ng/mL)	1.81 (1.02, 2.79)	2.00 (1.09, 3.19)	0.343	1.93 (1.09, 3.74)	1.96 (1.09, 3.05)	0.438	1.86 (1.02, 2.80)	1.97 (1.10, 3.18)	0.343
Adiponectin (ug/mL)	4.34 (2.83, 5.96)	4.81 (2.92, 7.59)	**0.015**	4.42 (2.85, 6.71)	4.75 (2.92, 7.45)	0.390	4.37 (2.79, 6.55)	4.82 (2.94, 7.61)	**0.006**
LAR	0.42 (0.20, 0.83)	0.41 (0.18, 0.85)	0.575	0.46 (0.21, 1.07)	0.41 (0.18, 0.83)	0.194	0.44 (0.21, 0.85)	0.40 (0.18, 0.84)	0.218
Resistin (ng/mL)	5.24 (4.16, 7.43)	5.00 (3.89, 6.67)	**0.009**	5.13 (4.00, 6.79)	5.05 (3.91, 6.74)	0.613	5.23 (4.05, 7.10)	5.02 (3.90, 6.68)	0.234
GlycA (mmol/L)	410.8 ± 68.9	408.8 ± 62.9	0.662	410.2 ± 57.1	408.9 ± 64.0	0.838	413.3 ± 76.1	408.3 ± 61.2	0.313
WBC (10^9^/L)	5.90 (5.00, 7.20)	5.70 (4.80, 6.70)	**0.044**	5.90 (4.70, 7.00)	5.70 (4.80, 6.80)	0.651	6.00 (4.90, 7.20)	5.70 (4.80, 6.70)	**0.031**
Monocytes (10^9^/L)	0.50 (0.42, 0.64)	0.50 (0.40, 0.62)	0.243	0.50 (0.42, 0.64)	0.50 (0.40, 0.62)	0.451	0.51 (0.42, 0.66)	0.50 (0.40, 0.61)	0.053
Basophils (10^9^/L)	0.033 (0.02, 0.04)	0.032 (0.02, 0.04)	0.253	0.033 (0.02, 0.04)	0.032 (0.02, 0.04)	0.286	0.035 (0.02, 0.04)	0.032 (0.02, 0.04)	**0.008**
Eosinophils (10^9^/L)	0.19 (0.12, 0.27)	0.17 (0.11, 0.25)	0.145	0.19 (0.12, 0.29)	0.17 (0.11, 0.25)	0.240	0.19 (0.12, 0.28)	0.17 (0.11, 0.25)	**0.038**
Neutrophils (10^9^/L)	3.16 (2.59, 4.06)	3.11 (2.51, 3.92)	0.314	3.18 (2.51, 3.96)	3.12 (2.52, 3.92)	0.749	3.22 (2.54, 4.21)	3.11 (2.52, 3.87)	0.066
Lymphocytes (10^9^/L)	1.88 (1.45, 2.36)	1.74 (1.42, 2.13)	**0.005**	1.77 (1.44, 2.24)	1.74 (1.43, 2.14)	0.755	1.74 (1.44, 2.17)	1.75 (1.43, 2.14)	0.597
NLR	1.75 (1.34, 2.23)	1.79 (1.41, 2.29)	0.178	1.76 (1.40, 2.22)	1.79 (1.40, 2.29)	0.746	1.79 (1.38, 2.40)	1.78 (1.40, 2.27)	0.629
PAI-1 (ng/mL)	28.3 ± 12.6	27.0 ± 12.2	0.155	27.8 ± 13.8	27.1 ± 12.2	0.527	28.3 ± 12.9	27.0 ± 12.1	0.098

Data are presented as mean (SD) or a median (IQR) for continuous variables and number and (%) for categorical variables. *p* for difference determined using an independent samples *t*-test or a Mann-Whitney *U* test. Significant *p* in **bold**.

C3: complement component 3; CRP: c-reactive protein; DASH: Dietary Approaches to Stop Hypertension; GlycA: glycoprotein acetyl; IL-6: interleukin 6; LAR: leptin-adiponectin ratio; NLR: neutrophil-lymphocyte ratio; PAI-1: plasminogen activator inhibitor-1; TNF-a: tumour necrosis factor alpha; WBC: white blood cell count.

Lower, more pro-inflammatory adiponectin concentrations and higher levels of resistin, WBCs and lymphocytes were observed in individuals with a history of childhood abuse (*p* = 0.015, *p* = 0.009, *p* = 0.044 and *p* = 0.005, respectively). There were no significant differences in inflammatory biomarkers levels according to exposure to neglect. Adiponectin concentrations were lower and WBC levels were higher in individuals who reported exposure to household dysfunction (*p* = 0.006 and *p* = 0.031, respectively). Examination of WBC constituents revealed higher concentrations of basophils (*p* = 0.008) and eosinophils (*p* = 0.038) among individuals exposed to household dysfunction.

### 3.3 Linear regression analysis

#### 3.3.1 ACE history

Results from linear regression analyses which investigated associations between ACE history and inflammatory biomarkers for the full sample are shown in Table 3. In crude models, having any ACE exposure was significantly associated with more pro-inflammatory concentrations of WBCs (*p* = 0.036), eosinophils (*p* = 0.031), lymphocytes (*p* = 0.035), PAI-1 (*p* = 0.037), and lower adiponectin levels (*p* = 0.002). In models which additionally adjusted for age, sex, education, anti-inflammatory medication use and morbidity (Model 3), associations with adiponectin (*p* = 0.032), eosinophil (*p* = 0.041) and PAI-1 *(p* = 0.046) concentrations remained, and CRP and resistin relationships with ACE history were also observed (*p* = 0.032 and *p* = 0.034, respectively). In fully adjusted models, any ACE exposure was significantly associated with lower concentrations of leptin (*p* = 0.024).

**Table 3:** Linear regression analysis of any ACE history and inflammatory biomarkers – full sample.

Biomarker	Model 1		Model 2		Model 3		Model 4	
	β (95% CI)	*p*	β (95% CI)	*p*	β (95% CI)	*p*	β (95% CI)	*p*
Log CRP	0.061 (−0.018, 0.139)	0.128	0.088 (0.010, 0.167)	**0.027**	0.086 (0.007, 0.164)	**0.032**	0.047 (−0.030, 0.124)	0.232
C3	0.638 (−2.055, 3.330)	0.642	0.978 (−1.736, 3.693)	0.480	0.857 (−1.820, 3.534)	0.53	−0.047 (−2.640, 2.546)	0.972
Log IL-6	0.022 (−0.061, 0.105)	0.608	0.068 (−0.014, 0.149)	0.104	0.055 (−0.025, 0.135)	0.18	0.020 (−0.062, 0.101)	0.636
Log TNF-α	0.001 (−0.037, 0.040)	0.954	0.016 (−0.022, 0.055)	0.403	0.016 (−0.023, 0.054)	0.422	0.018 (−0.023, 0.058)	0.387
Log leptin	−0.062 (−0.161, 0.038)	0.227	−0.038 (−0.135, 0.060)	0.448	−0.043 (−0.140, 0.054)	0.389	−0.097 (−0.182, −0.013)	**0.024**
Log adiponectin	−0.120 (−0.196, −0.045)	**0.002**	−0.076 (−0.143, −0.010)	**0.025**	−0.072 (−0.138, −0.006)	**0.032**	−0.045 (−0.112, 0.022)	0.186
LAR	0.058 (−0.068, 0.184)	0.364	0.039 (−0.088, 0.165)	0.549	−0.029 (−0.096, 0.154)	0.65	−0.053 (−0.161, 0.054)	0.332
Log resistin	0.048 (0.000, 0.096)	0.051	0.056 (0.007, 0.105)	**0.024**	0.052 (0.004, 0.100)	**0.034**	0.050 (−0.001, 0.100)	0.053
GlycA	1.938 (−5.130, 9.006)	0.591	4.870 (−2.059, 11.798)	0.168	4.669 (−2.236, 11.573)	0.185	3.028 (−4.092, 10.148)	0.404
Log WBC	0.033 (0.002, 0.063)	**0.036**	0.033 (0.002, 0.064)	**0.034**	0.029 (−0.001, 0.059)	0.058	0.013 (−0.016, 0.043)	0.375
Log monocytes	0.032 (−0.004, 0.068)	0.08	0.036 (0.001, 0.071)	**0.044**	0.031 (−0.004, 0.066)	0.078	0.020 (−0.016, 0.055)	0.277
Log basophils	0.059 (−0.003, 0.120)	0.063	0.059 (−0.003, 0.121)	0.064	0.057 (−0.006, 0.119)	0.076	0.056 (−0.009, 0.122)	0.09
Log eosinophils	0.075 (0.007, 0.144)	**0.031**	0.076 (0.007, 0.144)	**0.031**	0.071 (0.003, 0.140)	**0.041**	0.058 (−0.014, 0.129)	0.112
Log neutrophils	0.029 (−0.009, 0.066)	0.138	0.031 (−0.007, 0.068)	0.113	0.025 (−0.012, 0.062)	0.188	0.004 (−0.033, 0.041)	0.829
Log lymphocytes	0.039 (0.003, 0.075)	**0.035**	0.034 (−0.003, 0.070)	0.071	0.034 (−0.003, 0.070)	0.069	0.024 (−0.013, 0.061)	0.199
Log NLR	−0.010 (−0.054, 0.033)	0.639	−0.003 (−0.047, 0.040)	0.885	−0.009 (−0.052, 0.035)	0.689	−0.020 (−0.066, 0.026)	0.39
PAI-1	1.449 (0.091, 2.807)	**0.037**	1.377 (0.016, 2.739)	**0.047**	1.389 (0.026, 2.751)	**0.046**	1.223 (−0.191, 2.637)	0.09

Model 1: unadjusted; Model 2: adjusted for age and sex; Model 3: additionally adjusted for education, anti-inflammatory medication use, type 2 diabetes, cardiovascular disease and cancer; Model 4: additionally adjusted for smoking status, alcohol use, diet quality and BMI. Unstandardised β coefficients and 95% confidence intervals (CI) are shown. Significant *p* in **bold**.

C3: complement component 3; CRP: c-reactive protein; GlycA: glycoprotein acetyl; IL-6: interleukin 6; LAR: leptin-adiponectin ratio; NLR: neutrophil-lymphocyte ratio; PAI-1: plasminogen activator inhibitor-1; TNF-a: tumour necrosis factor alpha; WBC: white blood cell count.

#### 3.3.2 ACE subtypes

Table 4 presents findings from linear regression analyses of ACE subcategory associations with inflammatory biomarkers. In crude models, exposure to abuse was associated with higher resistin (*p* = 0.003), WBC (*p* = 0.043) and lymphocyte concentrations (*p* = 0.003) and lower adiponectin levels (*p* = 0.048). Associations with resistin and lymphocyte concentrations persisted upon full adjustment (*p* = 0.02 and *p* = 0.025, respectively), while negative associations with the NLR were also observed (*p* = 0.037).

**Table 4:** Linear regression analysis of ACE abuse, neglect and household dysfunction exposures and inflammatory biomarkers – full sample.

Biomarker	Model 1		Model 2		Model 3		Model 4	
Abuse exposure	β (95% CI)	*p*	β (95% CI)	*p*	β (95% CI)	*p*	β (95% CI)	*p*
Log CRP	0.001 (−0.099, 0.101)	0.98	0.028 (−0.072, 0.128)	0.582	0.015 (−0.084, 0.115)	0.765	−0.027 (−0.125, 0.071)	0.585
C3	1.186 (−2.248, 4.621)	0.498	1.513 (−1.936, 4.963)	0.390	0.968 (−2.433, 4.370)	0.577	−0.167 (−3.454, 3.120)	0.921
Log IL-6	0.014 (−0.092, 0.120)	0.798	0.062 (−0.042, 0.165)	0.244	0.037 (−0.065, 0.139)	0.48	0.013 (−0.091, 0.116)	0.811
Log TNF-α	0.009 (−0.040, 0.058)	0.713	0.025 (−0.024, 0.074)	0.312	0.020 (−0.029, 0.069)	0.424	0.024 (−0.027, 0.075)	0.361
Log leptin	−0.049 (−0.177, 0.078)	0.448	−0.028 (−0.153, 0.096)	0.654	−0.045 (−0.168, 0.078)	0.475	−0.103 (−0.210, 0.004)	0.06
Log adiponectin	−0.098 (−0.195, −0.001)	**0.048**	−0.057 (−0.142, 0.028)	0.187	−0.048 (−0.132, 0.036)	0.262	−0.027 (−0.112, 0.058)	0.534
Log LAR	0.047 (−0.114, 0.207)	0.569	0.028 (−0.133, 0.189)	0.732	0.002 (−0.157, 0.160)	0.982	−0.078 (−0.215, 0.058)	0.261
Log resistin	0.093 (0.032, 0.155)	**0.003**	0.101 (0.039, 0.163)	**0.001**	0.090 (0.029, 0.151)	**0.004**	0.076 (0.012, 0.139)	**0.02**
GlycA	2.013 (−7.009, 11.036)	0.662	4.740 (−4.077, 13.556)	0.292	3.799 (−4.982, 12.581)	0.396	1.831 (−7.205, 10.687	0.691
Log WBC	0.041 (0.001, 0.080)	**0.043**	0.041 (0.002, 0.080)	**0.038**	0.032 (−0.006, 0.070)	0.099	0.013 (−0.025, 0.051)	0.499
Log monocytes	0.033 (−0.014, 0.079)	0.168	0.037 (−0.007, 0.082)	0.1	0.029 (−0.015, 0.073)	0.2	0.016 (−0.029, 0.061)	0.491
Log basophils	0.051 (−0.028, 0.131)	0.207	0.051 (−0.029, 0.131)	0.211	0.046 (−0.034, 0.127)	0.256	0.038 (−0.045, 0.122)	0.368
Log eosinophils	0.069 (−0.019, 0.158)	0.126	0.070 (−0.018, 0.158)	0.117	0.060 (−0.028, 0.148)	0.18	0.039 (−0.052, 0.130)	0.396
Log neutrophils	0.028 (−0.020, 0.077)	0.252	0.031 (−0.017, 0.079)	0.21	0.019 (−0.028, 0.067)	0.428	−0.008 (−0.056, 0.039)	0.73
Log lymphocytes	0.070 (0.023, 0.116)	**0.003**	0.064 (0.017, 0.111)	**0.007**	0.060 (0.014, 0.106)	**0.011**	0.054 (0.007, 0.101)	**0.025**
Log NLR	−0.041 (−0.097, 0.015)	0.150	−0.033 (−0.089, 0.022)	0.241	−0.041 (−0.097, 0.015)	0.151	−0.062 (−0.121, −0.004)	**0.037**
PAI-1	1.260 (−0.475, 2.966)	0.155	1.200 (−0.534, 2.933)	0.175	1.128 (−0.606, 2.862)	0.202	0.772 (−1.022, 2.565)	0.399
**Neglect exposure**	**β (95% CI)**	***p***	**β (95% CI)**	***p***	**β (95% CI)**	***p***	**β (95% CI)**	***p***
Log CRP	0.073 (−0.060, 0.206)	0.28	0.095 (−0.038 (0.228)	0.161	0.073 (−0.059, 0.205)	0.28	−0.021 (−0.155, 0.113)	0.757
C3	2.080 (−2.473, 6.633)	0.37	2.118 (−2.446, 6.682)	0.363	1.574 (−2.925, 6.072)	0.493	−0.844 (−5.354, 3.666)	0.714
Log IL-6	0.126 (−0.015, 0.266)	0.081	0.193 (0.055, 0.331)	**0.006**	0.173 (0.037, 0.308)	**0.013**	0.088 (−0.054, 0.229)	0.224
Log TNF-α	0.015 (−0.050, 0.081)	0.649	0.037 (−0.028, 0.102)	0.269	0.030 (−0.034, 0.095)	0.356	0.021 (−0.049, 0.090)	0.563
Log leptin	0.090 (−0.079, 0.259)	0.295	0.073 (−0.091, 0.238)	0.382	0.067 (−0.097, 0.230)	0.424	−0.078 (−0.224, 0.069)	0.297
Log adiponectin	−0.058 (−0.187, 0.071)	0.377	−0.075 (−0.188, 0.037)	0.188	−0.069 (−0.180, 0.042)	0.226	−0.015 (−0.131, 0.102)	0.805
Log LAR	0.149 (−0.064, 0.362)	0.169	0.149 (−0.064, 0.362)	0.169	0.135 (−0.074, 0.345)	0.206	**−**0.063 (−0.249, 0.124)	0.509
Log resistin	0.039 (−0.043, 0.121)	0.353	0.040 (−0.042, 0.123)	0.337	0.032 (−0.050, 0.113)	0.445	0.004 (−0.084, 0.091)	0.932
GlycA	1.243 (−10.780, 13.285)	0.838	1.528 (−10.213, 13.270)	0.799	0.255 (−11,435, 11.946)	0.966	−2.776 (−15.209, 9.657)	0.662
Log WBC	0.012 (−0.040, 0.064)	0.651	0.019 (−0.032, 0.071)	0.459	0.012 (−0.038, 0.063)	0.63	−0.022 (−0.073, 0.030)	0.413
Log monocytes	0.020 (−0.041, 0.081)	0.523	0.042 (−0.017, 0.101)	0.161	0.035 (−0.023, 0.093)	0.234	0.015 (−0.046, 0.076)	0.634
Log basophils	0.052 (−0.052, 0.156)	0.329	0.050 (−0.055, 0.155)	0.346	0.048 (−0.058, 0.153)	0.375	0.040 (−0.073, 0.153)	0.488
Log eosinophils	0.065 (−0.052, 0.181)	0.275	0.082 (−0.034, 0.197)	0.167	0.076 (−0.039, 0.191)	0.198	0.28 (−0.095, 0.152)	0.651
Log neutrophils	0.010 (−0.054, 0.074)	0.766	0.021 (−0.042, 0.085)	0.512	0.013 (−0.049, 0.075)	0.681	−0.030 (−0.095, 0.035)	0.365
Log lymphocytes	0.009 (−0.053, 0.070)	0.782	0.001 (−0.061, 0.062)	0.981	−0.003 (−0.064, 0.058)	0.928	−0.028 (−0.092, 0.036)	0.393
Log NLR	0.001 (−0.073, 0.075)	0.978	0.021 (−0.053, 0.094)	0.583	0.016 (−0.057, 0.089)	0.67	−0.002 (−0.082, 0.087)	0.96
PAI-1	0.746 (−1,565, 3.057)	0.527	0.965 (−1.339, 3.270)	0.411	0.821 (−1.484, 3.125)	0.485	0.119 (−2.338, 2.475)	0.925
**Household dysfunction exposure**	**β (95% CI)**	***p***	**β (95% CI)**	***p***	**β (95% CI)**	***p***	**β (95% CI)**	***p***
Log CRP	0.086 (−0.006, 0.179)	0.067	0.122 (0.029, 0.215)	**0.01**	0.123 (0.031, 0.216)	**.009**	0.078 (−0.013, 0.169)	.092
C3	1.020 (−2.150, 4.189)	0.528	1.459 (−1.740, 4.658)	0.371	1.451 (−1.708, 4.609)	0.368	0.574 (−2.473, 3.622)	0.712
Log IL-6	0.071 (−0.027, 0.169)	0.154	0.132 (0.036, 0.229)	**0.007**	0.122 (0.027, 0.217)	**0.012**	0.090 (−0.006, 0.186)	0.066
Log TNF-α	0.011 (−0.034, 0.057)	0.63	0.031 (−0.014, 0.077)	0.178	0.032 (−0.013, 0.077)	0.162	0.031 (−0.016, 0.079)	0.197
Log leptin	−0.051 (−0.169, 0.067)	0.395	−0.020 (−0.138, 0.093)	0.703	−0.024 (−0.139, 0.091)	0.681	−0.082 (−0.182, 0.017)	0.104
Log adiponectin	−0.132 (−0.22, −0.042)	**0.004**	−0.080 (−0.158, −0.001)	**0.048**	−0.075 (−0.153, 0.003)	0.059	−0.049 (−0.128, 0.030)	0.222
Log LAR	0.082 (−0.067, 0.230)	0.281	0.058 (−0.092, 0.207)	0.448	0.052 (−0.096, 0.199)	0.493	−0.033 (−0.159, 0.094)	0.612
Log resistin	0.031 (−0.027, 0.088)	0.294	0.040 (−0.018, 0.097)	0.177	0.038 (−0.020, 0.095)	0.198	0.037 (−0.022, 0.096)	0.221
GlycA	5.009 (−3.337, 12.355)	0.239	8.644 (0.451, 16.836)	**0.039**	8.752 (0.581, 16.923)	**0.036**	6.234 (−2.149, 14.617)	0.145
Log WBC	0.046 (0.009, 0.082)	**0.013**	0.047 (0.011, 0.083)	**0.011**	0.043 (0.008, 0.079)	**0.017**	0.026 (−0.009, 0.061)	0.143
Log monocytes	0.046 (0.003, 0.089)	**0.034**	0.052 (0.011, 0.093)	**0.013**	0.047 (0.007, 0.088)	**0.023**	0.030 (−0.012, 0.071)	0.159
Log basophils	0.098 (0.025, 0.171)	**0.008**	0.099 (0.026, 0.173)	**0.008**	0.097 (0.023, 0.171)	**0.01**	0.087 (0.011, 0.164)	**0.026**
Log eosinophils	0.087 (0.006, 0.168)	**0.036**	0.088 (0.007, 0.169)	**0.033**	0.085 (0.004, 0.166)	**0.039**	0.084 (0.000, 0.167)	0.05
Log neutrophils	0.054 (0.009, 0.098)	**0.018**	0.057 (0.013, 0.102)	**0.012**	0.052 (0.008, 0.096)	**0.02**	0.030 (−0.014, 0.074)	0.181
Log lymphocytes	0.026 (−0.017, 0.069)	0.24	0.018 (−0.025, 0.062)	0.407	0.019 (−0.024, 0.062)	0.377	0.011 (−0.033, 0.054)	0.632
Log NLR	0.028 (−0.024, 0.080)	0.287	0.039 (−0.012, 0.091)	0.137	0.033 (−0.019, 0.084)	0.214	0.019 (−0.035, 0.073)	0.482
PAI-1	1.354 (−0.251, 2.960)	0.098	1.278 (−0.335, 2.890)	0.12	1.327 (−0.288, 2.942)	0.107	0.795 (−0.872, 2.461)	0.35

Model 1: unadjusted; Model 2: adjusted for age and sex; Model 3: additionally adjusted for education, anti-inflammatory medication use, type 2 diabetes, cardiovascular disease and cancer; Model 4: additionally adjusted for smoking status, alcohol use, diet quality and BMI. Unstandardised β coefficients and 95% confidence intervals (CI) are shown. Significant *p* in **bold**.

C3: complement component 3; CRP: c-reactive protein; GlycA: glycoprotein acetyl; IL-6: interleukin 6; LAR: leptin-adiponectin ratio; NLR: neutrophil-lymphocyte ratio; PAI-1: plasminogen activator inhibitor-1; TNF-a: tumour necrosis factor alpha; WBC: white blood cell count.

Neglect was associated with higher IL-6 levels (*p* = 0.013) (Model 3), but this relationship was attenuated in a fully adjusted model. In models which adjusted for age and sex (Model 2), the household dysfunction exposure was associated with a more pro-inflammatory profile than the other ACE subcategories, with relationships between household dysfunction and higher concentrations of CRP (*p* = 0.01) IL-6 (*p* = 0.007), GlycA (*p* = 0.039), WBCs (*p* = 0.011), monocytes (*p* = 0.013), basophils (*p* = 0.008), eosinophils (*p* = 0.033) and neutrophils (*p* = 0.012), and lower concentrations of adiponectin (*p* = 0.48) being observed. All of these associations (with the exception of adiponectin) persisted in Model 3. In a fully adjusted model, only household dysfunction relationships with higher basophil concentrations remained significant (*p* *=* 0.026).

#### 3.3.3 Sex-stratified analyses

Sex-stratified regression analyses which investigated associations between any ACE history and inflammatory biomarkers are shown in Supplementary Table 3. Among males, in age-adjusted analyses, ACE history was found to be significantly associated with higher concentration of TNF-a (*p* = 0.022), resistin (*p* = 0.04) and PAI-1 (*p* = 0.032). Associations remained significant for TNF-a (*p* = 0.017) and PA1-1 (*p* = 0.03) in fully adjusted models. Among female participants, age-adjusted analyses revealed associations between ACE history and lower adiponectin levels (*p* = 0.01) and higher concentrations of WBCs and constituents. Upon further adjustment for education, anti-inflammatory medication use, morbidity and lifestyle factors, relationships between any ACE history and WBC (*p* = 0.047), monocyte (*p* = 0.01) and eosinophil (*p* = 0.019) levels remained significant.

Sex-stratified linear regression analyses examining ACE subcategory associations with inflammatory biomarkers are presented in Supplementary Tables 4 and 5 Among male participants, in fully adjusted models, associations with lower leptin (*p* = 0.031) levels and the LAR (*p* = 0.041) were observed with the abuse subtype, while the neglect exposure was significantly related to lower concentrations of C3 (*p* = 0.004) and GlycA (p = 0.041). In partially adjusted models (Model 3), the household dysfunction subtype was related to higher concentrations of CRP (*p* = 0.007), TNF-α (*p* = 0.047) and PAI-1 (*p* = 0.007); associations with higher PAI-1 levels remained in Model 4 which additionally adjusted for lifestyle factors (*p* = 0.026). Among female participants, reported abuse exposure was significantly associated with higher WBC (*p* = 0.016), monocyte (*p* = 0.005) and lymphocyte (*p* = 0.003) levels after full adjustment, with the relationship between exposure to abuse and higher lymphocyte concentrations in female participants being the only association to withstand FDR correction in analyses (*p* = 0.048). No associations with any inflammatory biomarker were observed for the neglect subcategory in final models among women, while the household dysfunction subtype was found to be significantly associated with lower concentrations of leptin (*p* = 0.048) and higher levels of IL-6 (*p* = 0.043), monocytes (*p* = 0.032), basophils (*p* = 0.039), eosinophils (*p* = 0.006) and the NLR (*p* = 0.041) in fully adjusted models.

### 3.4 Mediation analysis

The results from mediation analyses which examined whether lifestyle factors mediate relationships between ACE history and inflammatory biomarkers concentrations are presented in Table 5. Among study participants who reported any ACE history, there was evidence that BMI mediates relationships between ACEs and higher concentrations of CRP, resistin and PAI-1, and lower levels of adiponectin, as indicated by a significant indirect effect (confidence intervals that did not include the null value) and/or a Sobel test *p* value less than 0.05). Similarly, analyses suggested that the association between the ACE neglect exposure and higher concentrations of IL-6 are mediated by BMI (indirect effect β = 0.045, 95% CI: 0.013, 0.082, Sobel test *p* = 0.002). For study participants who reported exposure to household dysfunction, smoking status was a significant mediator between household dysfunction and higher concentrations of IL-6, WBCs, monocytes and neutrophils, with findings suggesting that both smoking status and BMI mediate the relationship between household dysfunction and higher GlycA levels.

**Table 5:** Mediation analysis.

Any ACE history								
Biomarker	Mediator	Direct effect of ACE on biomarker		Indirect effect through mediator		Sobel test of mediation		Conclusion
		β	95% CI	β	95% CI	z	*p*	
Log CRP	Smoking status	0.085	0.006, 0.164	0.003	−0.001, 0.009	1.126	0.26	No mediation
	Alcohol use	0.084	0.006, 0.163	0.001	−0.003, 0.006	0.626	0.531	No mediation
	Diet quality	0.079	−0.002, 0.160	−0.001	−0.009, 0.006	−0.429	0.668	No mediation
	BMI	0.056	−0.019, 0.130	0.029	0.005, 0.055	2.321	0.02	**Mediation**
Log adiponectin	Smoking status	−0.077	−0.144, −0.011	−0.001	−0.004, 0.002	−0.506	0.613	No mediation
	Alcohol use	−0.074	−0.140, −0.008	0.002	−0.001, 0.008	1.180	0.238	No mediation
	Diet quality	−0.061	−0.130, 0.007	0.000	−0.002, 0.003	0.405	0.685	No mediation
	BMI	−0.052	−0.116, 0.011	−0.020	−0.038, −0.003	−2.306	0.021	**Mediation**
Log resistin	Smoking status	0.050	0.001, 0.099	0.000	−0.002, 0.003	0.465	0.642	No mediation
	Alcohol use	0.055	0.007, 0.103	−0.003	−0.007, 0.000	−1.536	0.124	No mediation
	Diet quality	0.054	0.004, 0.104	−0.000	−0.003, 0.002	−0.437	0.662	No mediation
	BMI	0.047	−0.001, 0.095	0.005	0.001, 0.011	2.004	0.045	**Mediation**
Log eosinophils	Smoking status	0.068	0.000, 0.136	0.006	−0.004, 0.018	1.106	0.269	No mediation
	Alcohol use	0.070	0.002, 0.139	0.001	−0.002, 0.006	0.709	0.478	No mediation
	Diet quality	0.067	−0.005, 0.139	−0.000	−0.005, 0.004	−0.234	0.815	No mediation
	BMI	0.069	0.001, 0.138	0.003	−0.001, 0.008	1.250	0.211	No mediation
PAI-1	Smoking status	1.371	−0.006, 2.749	0.086	−0.039, 0.251	1.260	0.208	No mediation
	Alcohol use	1.357	−0.007, 2.721	0.032	−0.035, 0.126	0.875	0.382	No mediation
	Diet quality	1.539	0.129, 2.950	−0.020	−0.119, 0.069	−0.462	0.644	No mediation
	BMI	1.178	−0.176, 2.533	0.215	0.032, 0.435	2.164	0.03	**Mediation**

Abuse exposure								
Biomarker	Mediator	Direct effect of ACE on biomarker		Indirect effect through mediator		Sobel test of mediation		Conclusion
		β	95% CI	β	95% CI	z	*p*	
Log resistin	Smoking status	0.089	0.027, 0.151	0.001	−0.003, 0.005	0.427	0.669	No mediation
	Alcohol use	0.091	0.030, 0.152	−0.001	−0.006, 0.002	−0.694	0.487	No mediation
	Diet quality	0.082	0.018, 0.145	−0.001	−0.004, 0.002	−0.505	0.613	No mediation
	BMI	0.084	0.023, 0.145	0.005	−0.001, 0.013	1.670	0.095	No mediation
Log lymphocytes	Smoking status	0.049	0.003, 0.059	0.009	−0.002, 0.020	1.539	0.124	No mediation
	Alcohol use	0.060	0.013, 0.106	0.000	−0.001, 0.002	0.412	0.68	No mediation
	Diet quality	0.070	0.022, 0.118	−0.001	−0.005, 0.002	−0.653	0.514	No mediation
	BMI	0.057	0.011, 0.103	0.003	−0.001, 0.008	1.386	0.166	No mediation

Neglect exposure								
Biomarker	Mediator	Direct effect of ACE on biomarker		Indirect effect through mediator		Sobel test of mediation		Conclusion
		β	95% CI	β	95% CI	z	*p*	
Log IL-6	Smoking status	0.181	0.044, 0.319	0.000	−0.018, 0.019	0.003	0.998	No mediation
	Alcohol use	0.175	0.040, 0.310	−0.002	−0.011, 0.006	−0.583	0.56	No mediation
	Diet quality	0.118	−0.026, 0.262	−0.002	−0.018, 0.013	−0.311	0.756	No mediation
	BMI	0.128	−0.005, 0.262	0.045	0.013, 0.082	3.026	0.002	**Mediation**

Household dysfunction exposure								
Biomarker	Mediator	Direct effect of ACE on biomarker		Indirect effect through mediator		Sobel test of mediation		Conclusion
		β	95% CI	β	95% CI	z	*p*	
Log CRP	Smoking status	0.120	0.027, 0.213	0.005	0.000, 0.014	1.546	0.122	No mediation
	Alcohol use	0.122	0.029, 0.214	0.002	−0.004, 0.009	0.588	0.556	No mediation
	Diet quality	0.113	0.018, 0.208	−0.004	−0.013, 0.004	−0.932	0.351	No mediation
	BMI	0.095	0.007, 0.183	0.028	−0.001, 0.058	1.899	0.059	No mediation
Log IL-6	Smoking status	0.111	0.015, 0.206	0.014	0.002, 0.029	2.124	0.034	**Mediation**
	Alcohol use	0.115	0.020, 0.211	0.007	0.000, 0.016	1.678	0.093	No mediation
	Diet quality	0.126	0.029, 0.224	−0.005	−0.017, 0.005	−0.960	0.337	No mediation
	BMI	0.103	0.010, 0.197	0.020	0.000, 0.040	1.934	0.053	No mediation
GlycA	Smoking status	7.878	−0.271, 16.028	1.441	0.244, 2.925	2.161	0.031	**Mediation**
	Alcohol use	8.210	0.033, 16.388	0.541	−0.007, 1.399	1.642	0.101	No mediation
	Diet quality	8.600	0.123, 17.077	−0.243	−0.964, 0.364	−0.802	0.423	No mediation
	BMI	7.910	−0.238, 16.058	0.858	0.029, 1.979	1.848	0.065	**Mediation**
Log WBC	Smoking status	0.034	−0.001, 0.068	0.010	0.001, 0.021	2.012	0.044	**Mediation**
	Alcohol use	0.042	0.007, 0.078	0.001	−0.002, 0.004	0.685	0.493	No mediation
	Diet quality	0.044	0.008, 0.080	−0.002	−0.008, 0.003	−0.847	0.397	No mediation
	BMI	0.039	0.003, 0.074	0.005	0.000, 0.010	1.816	0.069	No mediation
Log monocytes	Smoking status	0.039	−0.002, 0.079	0.009	0.000, 0.019	1.997	0.046	**Mediation**
	Alcohol use	0.044	0.003, 0.085	0.003	0.000, 0.008	1.854	0.064	No mediation
	Diet quality	0.048	0.006, 0.090	−0.002	−0.008, 0.003	−0.853	0.394	No mediation
	BMI	0.045	0.004, 0.086	0.002	0.000, 0.006	1.554	0.120	No mediation
Log basophils	Smoking status	0.093	0.020, 0.167	0.010	0.000, 0.022	1.879	0.06	No mediation
	Alcohol use	0.094	0.020, 0.168	0.003	−0.002, 0.009	1.093	0.274	No mediation
	Diet quality	0.098	0.022, 0.174	−0.001	−0.006, 0.002	−0.801	0.423	No mediation
	BMI	0.098	0.024, 0.172	−0.001	−0.005, 0.003	−0.466	0.641	No mediation
Log eosinophils	Smoking status	0.078	−0.002, 0.158	0.013	0.000, 0.027	1.898	0.058	No mediation
	Alcohol use	0.083	0.002, 0.164	0.002	−0.003, 0.008	0.701	0.483	No mediation
	Diet quality	0.101	0.017, 0.185	−0.002	−0.008, 0.003	−0.813	0.416	No mediation
	BMI	0.083	0.002, 0.164	0.003	−0.001, 0.009	1.213	0.225	No mediation
Log neutrophils	Smoking status	0.044	0.001, 0.087	0.010	0.001, 0.020	2.046	0.041	**Mediation**
	Alcohol use	0.052	0.008, 0.096	0.000	−0.003, 0.004	0.152	0.879	No mediation
	Diet quality	0.048	0.003, 0.093	−0.003	−0.011, 0.044	−0.857	0.391	No mediation
	BMI	0.046	0.002, 0.089	0.006	0.000, 0.014	1.852	0.064	No mediation

All models adjusted for age, sex. education, anti-inflammatory medication use, type 2 diabetes, cardiovascular disease and cancer. CRP: c-reactive protein; GlycA: glycoprotein acetyl; IL-6: interleukin 6; plasminogen activator inhibitor-1; WBC: white blood cell count. Unstandardised β coefficients are shown. 95% confidence intervals (CI) determined from 5000 bootstrap samples.

## 4 Discussion

This study investigated ACE history and ACE subcategory relationships with a range of inflammatory biomarkers in a middle-to older-aged Irish population for the entire sample and stratified by sex. With regard to ACE history, almost 23% of participants in our sample reported exposure to any ACE; this is comparable to findings from the Irish Longitudinal Study on Ageing (TILDA), a nationally representative study, where 26% of subjects reported an ACE exposure (Ward et al., 2020). Our results demonstrate associations between reported ACE exposure and more pro-inflammatory and pro-thrombotic profiles in adulthood, which were ACE subtype and sex specific. Examination of ACE subtypes indicated that associations appear to be driven by household dysfunction, as this subcategory was associated with a more pro-inflammatory profile than abuse or neglect through more pro-inflammatory concentrations of CRP, IL-6, GlycA, WBCs, and WBC constituents, and lower adiponectin levels. These relationships were robust to adjustment for age and sex, education, anti-inflammatory medication use and chronic disease history, but all were attenuated, except for basophil concentrations, in models that accounted for lifestyle behaviours (smoking status, alcohol use, diet quality) and BMI. Sex-stratified analyses revealed that significant associations between ACE subcategories and higher concentrations of inflammatory biomarkers are driven primarily by female participants. Mediation analyses suggested that lifestyle factors, specifically BMI and smoking status, mediate relationships between ACE exposures and certain biomarkers. Thus, these data provide valuable insights into the potential relationship between childhood adversity and pro-inflammatory profiles in adulthood.

Increased inflammation has been previously observed in adults who have experienced childhood trauma (Bertone-Johnson et al., 2012; Crick et al., 2022; Kiecolt-Glaser et al., 2011; Kraynak et al., 2019; Lacey et al., 2020; Smith et al., 2011) and these unfavourable inflammatory profiles are associated with increased cardiometabolic risk (Ridker et al., 2023). In our research, regression analyses revealed associations between exposure to any ACE and increased CRP, resistin, eosinophils, PAI-1, and decreased adiponectin concentrations in partially adjusted models. Among previous studies examining ACE history and adult immune status, one study found significant positive associations with lymphocytes levels, but significant associations were not found for WBCs, granulocytes or monocytes (Surtees et al., 2003), while another study found higher total WBCs (Etzel et al., 2022). Findings for adiponectin are consistent with previously reported negative associations with ACE exposure (Tietjen et al., 2012), which is expected, as lower adiponectin levels are more pro-inflammatory. Regarding PAI-1, this study presents novel findings, as PAI-1 has not yet been studied in healthy adults in relation to ACEs.

Examination of ACE subcategories revealed that, in partially adjusted models, exposure to abuse was associated with higher resistin and lymphocyte concentrations while exposure to neglect was associated with higher levels of IL-6. Childhood household dysfunction, the most prevalent ACE subtype reported in this sample, was associated with higher concentrations of CRP, IL-6, GlycA, WBCs, and WBC constituents, in partially adjusted models. This is consistent with results found in previous research by our group which showed that exposure to childhood household dysfunction was associated with unfavourable high-density lipoprotein cholesterol and triglyceride concentrations and pro-atherogenic indices, suggesting increased cardiometabolic risk in adulthood (O'Leary et al., 2023). Therefore, household dysfunction may be a key driver of associations between exposure to any ACE and greater inflammation in adulthood. However, this contrasts with previous studies which have identified childhood abuse, including emotional, physical and sexual abuse, as the strongest predictor of chronic inflammation in adulthood (Kiecolt-Glaser et al., 2011; Kraynak et al., 2019; Pereira et al., 2019; Smith et al., 2011). It should be noted that these studies did not fully survey participants for exposure to household dysfunction, and only asked about death in the family and/or divorce, potentially explaining discrepancies.

Importantly, many significant associations between ACE exposures and inflammatory biomarkers were attenuated after full adjustment for lifestyle behaviours and BMI. This is consistent with previous research and implies that factors related to ACEs (i.e. smoking status, alcohol use, diet quality and overweight and obesity) may partly explain associations between ACE history and systemic inflammation in adulthood (Chen & Lacey, 2018; Kuzminskaite et al., 2020; Rooks et al., 2012). Mediation analyses suggested that BMI and smoking status mediate relationships between ACEs and inflammatory biomarker concentrations. Notably, BMI and smoking status differed significantly based on exposure to any ACE and exposure to abuse. Among individuals with exposure to neglect, only BMI was significantly higher, and among those who reported exposure to household dysfunction, only smoking status was significantly higher. Individuals with a history of childhood trauma are often more likely to engage in health risk behaviours such as smoking, drinking and use of illicit drugs (Dube et al., 2003; Su et al., 2015) and are at higher risk of obesity (Baldwin & Danese, 2019), which contribute to increased inflammation (Millar, et al., 2022). It is important to note that 18% of Irish people aged 15 years and older smoke (Tobacco Free Ireland Programme, 2022) and that Ireland ranks high among countries with the greatest alcohol consumption and prevalence of binge drinking (Health Research Board, 2021). Ireland also has some of the highest rates of obesity in Europe, with 60% of Irish adults being overweight or obese (Health Service Executive, 2023).

Given results from mediation analyses and evidence from previous studies, it is possible that higher BMI and former or current smoking status are both consequences of exposure to ACEs and contributors to increased inflammation in adulthood. These findings would support an accumulation of risk model of understanding the relationship between ACEs and CVD, where exposure to traumatic events in childhood may lead to obesity and substance misuse across the life-course, contributing to increased inflammation and risk of CVD. It should be noted, however, that some relationships withstood adjustment for lifestyle behaviours and BMI, with associations between any ACE history and increased TNF-α and PAI-1 levels persisting among males, while relationships with WBCs and WBC constituents persisted among females.

This study has several strengths. As far as we are aware, this research is the first to assess ACE history and ACE subcategory and sex-specific relationships with a wide range of markers of chronic low-grade inflammation and raised immune activation in a middle-to older-aged population. Therefore, our study has examined the greatest number of biomarkers in a relatively large population in this context. Other strengths include equal representation by sex (49% male) and similar age and sex demographics between the analytic sample and the entire cohort (data not shown). This study also used a validated questionnaire to define ACE history.

A potential limitation is that ACE history (coded 0–8) was examined in regression analyses as a binary variable; while this classification is consistent with previous studies (Cheong et al., 2017; O'Leary et al., 2023), it does not account for variation in level of ACE. Studies have identified dose-dependent relationships between ACEs and inflammation levels, suggesting multiple ACE exposures may have a cumulative effect on CVD risk (Bertone-Johnson et al., 2012; Iob et al., 2020). Therefore, it is possible that results may change slightly depending on how ACE is categorised. It should be noted that self-reported questionnaires, such as the ACE questionnaire, are subject to potential inaccuracies and recall and reporting bias, especially given the age demographics of this cohort that spans from midlife to later life. The eldest participants in the study are self-reporting ACEs in the context of cultural and parenting norms that were different from the youngest participants. The ability to recall events during childhood is also potentially hindered with age for the oldest portion of this cohort.

While our regression analyses controlled for a range of potential confounders, other unknown or unmeasured factors could be considered. Notably, although we adjusted for education in analyses, we did not have data on socio-economic status for our study sample; consequently, the possibility of residual confounding should be considered. Additionally, ‘inflammageing’, a condition where older adults display increased levels of inflammatory biomarkers and progressively increasing CVD risk, may distort the relationship between ACEs and inflammatory biomarkers (Ferrucci & Fabbri, 2018). However, given the makeup of this cohort, dividing participants by age would result in arbitrary categories with unequal numbers of participants in each, so we attempt to mitigate these effects by controlling for age as a continuous variable in regression analyses.

The nature of this study did not allow for detailed pre-specification of hypotheses and, as we examined a large number of biomarkers, the risk of type I errors is a possibility. While we address this using a stringent Romano-Wolf multiple hypothesis correction (Clarke et al., 2019), it should be noted that although correcting for multiple comparisons reduces the probability of false significant findings, it also increases the probability of false negative results. Replication of these findings in future work would contribute to their robustness. Finally, the generalisability of our findings may be limited. Ireland represents a generally ethnically homogeneous population (Cronin et al., 2008). Previous research suggests that approximately 98% of Irish adults are registered with a GP and that, even in the absence of a universal patient registration system, it is possible to perform population-based epidemiological studies that are representative using our methods (Hinchion et al., 2002). However, our data were collected from a single primary care-based sample which may not be representative of the general population and, therefore, further examination in other populations is suggested.

In conclusion, results from this research contribute unique data on the relationship between childhood adversity and later life cardiometabolic risk with potential to deepen our theoretical understanding of causal and mediating factors. We found that exposure to ACEs, reported in almost 23% of study participants, is associated with more pro-inflammatory and pro-thrombotic profiles, with evidence of ACE subtype and sex-specific associations. Mediation analyses suggested that lifestyle factors, specifically BMI and smoking status, mediate relationships between ACE exposures and certain inflammatory biomarkers. Thus, it is arguable that adjusting for lifestyle behaviours when studying relationships between ACEs and disease biomarkers may represent an over-adjustment that conceals important causal effects. Therefore, further research on ACEs and chronic inflammation, which considers diverse and vulnerable populations, sex differences and ACE subtypes, other potential confounders and the possible mediating role of lifestyle factors and obesity, is warranted.

## Source of support

This work was supported by a research grant from the Irish Health Research Board (reference: HRC/2007/13). The funders had no role in the study design, data collection and analysis, decision to publish or preparation of the manuscript.

## Ethical statement

Ethics committee approval conforming to the Declaration of Helsinki was obtained from the Clinical Research Ethics Committee of University College Cork.

## Declaration of competing interests

None declared.

## CRediT authorship contribution statement

**Caroline Pitts:** Conceptualization, Investigation, Writing – original draft, Writing – review & editing. **Seán R. Millar:** Formal analysis, Resources, Writing – review & editing. **Ivan J. Perry:** Funding acquisition, Project administration, Resources, Writing – review & editing. **Catherine M. Phillips:** Conceptualization, Funding acquisition, Investigation, Project administration, Resources, Supervision, Writing – original draft, Writing – review & editing.

## Data Availability

Data will be made available on request.

## References

[bib1] American Diabetes Association (2014). Diagnosis and classification of diabetes mellitus. Diabetes Care.

[bib2] Anda R.F., Butchart A., Felitti V.J., Brown D.W. (2010). Building a framework for global surveillance of the public health implications of adverse childhood experiences. American Journal of Preventative Medicine.

[bib3] Baldwin J.R., Danese A. (2019). Pathways from childhood maltreatment to cardiometabolic disease: A research review. CoramBAAF Adoption and Fostering Academy.

[bib4] Ben-Shlomo Y., Kuh D. (2002). A life course approach to chronic disease epidemiology: Conceptual models, empirical challenges and interdisciplinary perspectives. International Journal of Epidemiology.

[bib5] Bertone-Johnson E.R., Whitcomb B.W., Missmer S.A., Karlson E.W., Rich-Edwards J.W. (2012). Inflammation and early-life abuse in women. American Journal of Preventative Medicine.

[bib6] Bingham S.A., Gill C., Welch A., Cassidy A., Runswick S.A., Oakes S. (1997). Validation of dietary assessment methods in the UK arm of EPIC using weighed records, and 24-hour urinary nitrogen and potassium and serum vitamin C and carotenoids as biomarkers. International Journal of Epidemiology.

[bib7] Cable N. (2014). Life course approach in social epidemiology: An overview, application, and future implications. Journal of Epidemiology.

[bib8] Campbell J.A., Walker R.J., Egede L.E. (2015). Associations between adverse childhood experiences, high-risk behaviors, and morbidity in adulthood. American Journal of Preventative Medicine.

[bib9] Carpenter L.L., Gawuga C.E., Tyrka A.R., Lee J.K., Anderson G.M., Price L.H. (2010). Association between plasma IL-6 response to acute stress and early-life adversity in healthy adults. Neuropsychopharmacology.

[bib10] Carroll J.E., Gruenewald T.L., Taylor S.E., Janicki-Deverts D., Matthews K.A., Seemanf T.E. (2013). Childhood abuse, parental warmth, and adult multisystem biological risk in the Coronary Artery Risk Development in Young Adults study. Proceedings of the National Academy of Sciences of the USA.

[bib11] Chen M., Lacey R.E. (2018). Adverse childhood experiences and adult inflammation: Findings from the 1958 British birth cohort. Brain, Behavior, and Immunity.

[bib12] Cheong E.V., Sinnott C., Dahly D., Kearney P.M. (2017). Adverse childhood experiences (ACEs) and later-life depression: Perceived social support as a potential protective factor. BMJ Open.

[bib13] Chiesa S.T., Charakida M., Georgiopoulos G., Roberts J.D., Stafford S.J., Park C., Mykkänen J., Kähönen M., Lehtimäki T., Ala-Korpela M., Raitakari O., Pietiäinen M., Pussinen P., Muthurangu V., Hughes A.D., Sattar N., Timpson N.J., Deanfield J.E. (2022). Glycoprotein acetyls: A novel inflammatory biomarker of early cardiovascular risk in the young. Journal of the American Heart Associaton.

[bib14] Clarke D., Romano J.P., Wolf M. (2019). The romano-wolf multiple hypothesis correction in Stata. STATA Journal.

[bib15] Crick D.C.P., Halligan S.L., Howe L.D., Lacey R.E., Khandaker G.M. (2022). Associations between Adverse Childhood Experiences and the novel inflammatory marker glycoprotein acetyls in two generations of the Avon Longitudinal Study of Parents and Children birth cohort. Brain, Behavior, and Immunity.

[bib16] Cronin S., Berger S., Ding J., Schymick J.C., Washecka N. (2008). A genome-wide association study of sporadic ALS in a homogenous Irish population. Human Molecular Genetics.

[bib17] Dube S.R., Felitti V.J., Dong M., Chapman D.P., Giles W.H., Anda R.F. (2003). Childhood abuse, neglect, and household dysfunction and the risk of illicit drug use: The adverse childhood experiences study. Pediatrics.

[bib18] Etzel L., Apsley A., Mattern B.C., Hastings W.J., Heller T., Ram N., Siegel S.R., Shalev I. (2022). Immune cell dynamics in response to an acute laboratory stressor: A within-person between-group analysis of the biological impact of early life adversity. Stress: The International Journal on the Biology of Stress.

[bib19] Ferrucci L., Fabbri E. (2018). Inflammageing: Chronic inflammation in ageing, cardiovascular disease, and frailty. Nature Reviews Cardiology.

[bib20] Friel S., Nic Gabhainn S., Kelleher C. (1997).

[bib21] Fruhbeck G., Catalan V., Rodriguez A., Gomez-Ambrosi J. (2018). Adiponectin-leptin ratio: A promising index to estimate adipose tissue dysfunction. Relation with obesity-associated cardiometabolis risk. Adipocyte.

[bib22] Giano Z., Wheeler D.L., Hubach R.D. (2020). The frequencies and disparities of adverse childhood experiences in the U.S. BMC Public Health.

[bib23] Haahr-Pedersen I., Perera C., Hyland P., Vallieres F., Murphy D., Hansen M., Spitz P., Hansen P., Cloitre M. (2020). Females have more complex patterns of childhood adversity: Implications for mental, social, and emotional outcomes in adulthood. European Journal of Psychotraumatology.

[bib24] Harrington J. (1997).

[bib25] Harrington J.M., Fitzgerald A.P., Kearney P.M., McCarthy V.J., Madden J., Browne G. (2013). DASH diet score and distribution of blood pressure in middle-aged men and women. American Journal of Hypertension.

[bib26] Hayes A.F. (2017).

[bib27] Health Research Board (2021). https://www.hrb.ie/fileadmin/2._Plugin_related_files/Publications/2021_publications/2021_HIE/Evidence_Centre/HRB_Alcohol_Overview_Series_11.pdf.

[bib28] Health Service Executive (2023). https://www.hse.ie/eng/about/who/cspd/ncps/obesity/.

[bib29] Hinchion R., Sheehan J., Perry I. (2002). Primary care research: Patient registration. Irish Medical Journal.

[bib30] Hostinar C.E., Lachman M.E., Mroczek D.K., Seeman T.E., Miller G.E. (2015). Additive contributions of childhood adversity and recent stressors to inflammation at midlife: Findings from the MIDUS study. Developmental Psychology.

[bib31] Iob E., Lacey R., Steptoe A. (2020). The long-term association of adverse childhood experiences with C-reactive protein and hair cortisol: Cumulative risk versus dimensions of adversity. Brain, Behavior, and Immunity.

[bib32] Kearney P.M., Harrington J.M., McCarthy V.J., Fitzgerald A.P., Perry I.J. (2013). Cohort profile: The Cork and Kerry diabetes and Heart disease study. International Journal of Epidemiology.

[bib33] Kelleher C., Nic Gabhainn S., Friel S., Corrigan H., Nolan G., Sixsmith J. (2003).

[bib34] Kiecolt-Glaser J.K., Gouin J.-P., Weng N.-p., Malarkey W.B., Beversdorf D.Q., Glaser R. (2011). Childhood adversity heightens the impact of later-life caregiving stress on telomere length and inflammation. Psychosomatic Medicine.

[bib35] Kraynak T., Marsland A.L., Hanson J.L., Gianaros P.J. (2019). Retrospectively reported childhood physical abuse, systemic inflammation, and resting corticolimbic connectivity in midlife adults. Brain, Behavior, and Immunity.

[bib36] Kuzminskaite E., Vinkers C.H., Elzinga B.M., Wardenaar K.J., Giltay E.J., Penninx B.W.J.H. (2020). Childhood trauma and dysregulation of multiple biological stress systems in adulthood: Results from The Netherlands Study of Depression and Anxiety (NESDA). Psychoneuroendocrinology.

[bib37] Lacey R.E., Pereira S.M.P., Li L., Danese A. (2020). Adverse childhood experiences and adult inflammation: Single adversity, cumulative risk and latent class approaches. Brain, Behavior, and Immunity.

[bib38] Millar S.R., Harrington J.M., Perry I.J., Phillips C.M. (2022). Associations between a protective lifestyle behaviour score and biomarkers of chronic low-grade inflammation: A cross-sectional analysis in middle-to-older aged adults. International Journal of Obesity.

[bib39] Mize T.D. (2023). Sobel-Goodman test of mediation in stata.

[bib40] Morgan K., McGee H., Watson D., Perry I., Barry M.M., Shelley E. (2008).

[bib41] O'Leary E., Millar S.R., Perry I.J., Phillips C.M. (2023). Association of adverse childhood experiences with lipid profiles and atherogenicrisk indices in a middle-to-older aged population. SSM - Population Health.

[bib42] Otvos J., Shalaurova I., Wolak-Dinsmore J., Connelly M., Mackey R., Stein J., Tracy R. (2015). GlycA: A composite nuclear magnetic resonance biomarker of systemic inflammation. Clinical Chemistry.

[bib43] Pereira S.M.P., Merkin S.S., Seeman T., Power C. (2019). Understanding associations of early-life adversities with mid-life inflammatory profiles: Evidence from the UK and USA. Brain, Behavior, and Immunity.

[bib44] Petruccelli K., Davis J., Berman T. (2019). Adverse childhood experiences and associated health outcomes: A systematic review and meta-analysis. Child Abuse & Neglect.

[bib45] Phillips C.M., Dillon C.B., Perry I.J. (2017). Does replacing sedentary behaviour with light or moderate to vigorous physical activity modulate inflammatory status in adults?. International Journal of Behavioral Nutrition and Physical Activity.

[bib46] Randox Biosciences (2023). https://www.randox.com/biosciences-overview/.

[bib47] Riboli E., Elmåhl S., Saracci R., Gullberg B., Lingärde F. (1997). The malmö food study: Validity of two dietary assessment methods for measuring nutrient intake. International Journal of Epidemiology.

[bib48] Ridker P.M., Bhatt D.L., Pradhan A.D., Glynn R.J., MacFadyen J.G., Nissen S.E. (2023). Inflammation and cholesterol as predictors of cardiovascular events among patients receiving statin therapy: A collaborative analysis of three randomised trials. The Lancet.

[bib49] Rooks C., Veledar E., Goldberg J., Bremner J.D., Vaccarino V. (2012). Early trauma and inflammation: Role of familial factors in a study of twins. Psychosomatic Medicine.

[bib50] Scott J., McMillian-Bohler J., Johnson R., Simmons L.A. (2021). Adverse childhood experiences and blood pressure in women in the United States: A systematic review. Journal of Midwifery & Women's Health.

[bib51] Smith A.K., Conneely K.N., Kilaru V., Mercer K.B., Weiss T.E., Bradley B., Tang Y., Gillespie C.F., Cubells J.F., Ressler K.J. (2011). Differential immune system DNA methylation and cytokine regulation in post-traumatic stress disorder. American Journal of Medical Genetics Part B: Neuropsychiatric Genetics.

[bib52] Sokol A., Wirth M.D., Manczuk M., Shivappa N., Zatonska K., Hurley T.G. (2016). Association between the dietary inflammatory index, waist-to-hip ratio and metabolic syndrome. Nutrition Research.

[bib53] Su S., Jimenez M.P., Roberts C.T.F., Loucks E.B. (2015). The role of adverse childhood experiences in cardiovascular disease risk: A review with emphasis on plausible mechanisms. Current Cardiology Reports.

[bib54] Suglia S.F., Koenen K.C., Boynton-Jarrett R., Chan P.S., Clark C.J., Danese A., Faith M.S., Goldstein B.I., Hayman L.L., Isasi C.R., Pratt C.A., Slopen N., Sumner J.A., Turer A., Turer C.B., Zacharia J.P. (2017). Childhood and adolescent adversity and cardiometabolic outcomes: A scientific statement from the American Heart associaton. Circulation.

[bib55] Surtees P., Wainwright N., Day N., Brayne C., Luben R., Khaw K.-T. (2003). Adverse experience in childhood as a developmental risk factor for altered immune status in adulthood. International Journal of Behavioral Medicine.

[bib56] Tanaka T., Narazaki M., Kishimoto T. (2014). IL-6 in inflammation, immunity, and disease. Cold Spring Harbor Perspectives in Biology.

[bib57] Tietjen G.E., Khubchandani J., Herial N.A., Shah K. (2012). Adverse childhood experiences are associated with migraine and vascular biomarkers. Headache.

[bib58] Tobacco Free Ireland Programme (2022). https://www.hse.ie/eng/about/who/tobaccocontrol/news/state-of-tobacco-control-report2022.pdf.

[bib59] Ward M., Turner N., Briggs R., O'Halloran A.M., Kenny R.A. (2020). Resilience does not mediate the association between adverse childhood experiences and later life depression. Findings from the Irish Longitudinal Study on Ageing (TILDA). Journal of Affective Disorders.

[bib60] World Health Organization (2019). https://www.who.int/news-room/fact-sheets/detail/cardiovascular-diseases-(cvds.

[bib61] Zhu S., Shan S., Liu W., Li S., Hou L., Huang X., Liu Y., Yi Q., Sun W., Tang K., Adeloye D., Rudan I., Song P. (2022).

