# Supplementary material for: Relationships between childhood adversity and inflammatory biomarkers in adulthood: A cross-sectional analysis of a middle-to older-aged population
Source: SSM Popul Health. 2024 Jan 12;25:101608. doi: 10.1016/j.ssmph.2024.101608 (PMC10797532; doi:10.1016/j.ssmph.2024.101608)
Supplement: Multimedia component 1 [file mmc1.docx]

| **Supplementary Table 1.** ACE comparative statistics – full sample and by sex | | | | | |
| --- | --- | --- | --- | --- | --- |
| **Characteristic** | **Level** | **All** | **Sex** | | ***p*** |
|  |  | **(n=1839)** | **Male**  **(n=904)** | **Female (n=935)** |  |
| **Any ACE history** |  |  |  |  | 0.2 |
| Yes | n (%) | 416 (22.6) | 216 (23.9) | 200 (21.4) |  |
| No | n (%) | 1423 (77.4) | 688 (76.1) | 735 (78.6) |  |
|  |  |  |  |  |  |
| **Abuse exposure** |  |  |  |  | 0.36 |
| Yes | n (%) | 225 (12.2) | 117 (12.9) | 108 (11.6) |  |
| No | n (%) | 1614 (87.8) | 787 (87.1) | 827 (88.4) |  |
|  |  |  |  |  |  |
| **Neglect exposure** |  |  |  |  | 0.22 |
| Yes | n (%) | 119 (6.5) | 52 (5.8) | 67 (7.2) |  |
| No | n (%) | 1720 (93.5) | 852 (94.2) | 868 (92.8) |  |
|  |  |  |  |  |  |
| **Household dysfunction exposure** |  |  |  |  | 0.2 |
| Yes | n (%) | 271 (14.7) | 143 (15.8) | 128 (13.7) |  |
| No | n (%) | 1568 (85.3) | 761 (84.2) | 807 (86.3) |  |
|  |  |  |  |  |  |
| Data are presented as number and (%); *p* determined from a chi-square test. | | | | | |

| **Supplementary Table 2**. Individual ACE questionnaire items – full sample and by sex | | | | |
| --- | --- | --- | --- | --- |
| **Characteristic** | **Level** | **All** | **Sex** | |
|  |  | **(n=1839)** | **Male**  **(n=904)** | **Female (n=935)** |
| ***Abuse*** |  |  |  |  |
| **Parent/adult threatened you** |  |  |  |  |
| Yes | n (%) | 145 (7.9) | 74 (8.2) | 71 (7.6) |
| **Parent/adult hit you** |  |  |  |  |
| Yes | n (%) | 120 (6.5) | 60 (6.6) | 60 (6.4) |
| **Adult sexually abused you** |  |  |  |  |
| Yes | n (%) | 77 (4.2) | 37 (4.1) | 40 (4.3) |
| ***Neglect*** |  |  |  |  |
| **Felt unloved by family** |  |  |  |  |
| Yes | n (%) | 112 (6.1) | 48 (5.3) | 64 (6.8) |
| **Not enough to eat/neglected** |  |  |  |  |
| Yes | n (%) | 25 (1.4) | 10 (1.1) | 15 (1.6) |
| ***Household dysfunction*** |  |  |  |  |
| **Parents ever separated/divorced** |  |  |  |  |
| Yes | n (%) | 49 (2.7) | 24 (2.7) | 25 (2.7) |
| **Mother/stepmother beaten** |  |  |  |  |
| Yes | n (%) | 41 (2.2) | 18 (2.0) | 23 (2.5) |
| **Lived with problem drinker/drug addict** |  |  |  |  |
| Yes | n (%) | 127 (6.9) | 60 (6.6) | 67 (7.2) |
| **Household member was depressed** |  |  |  |  |
| Yes | n (%) | 132 (7.2) | 76 (8.4) | 56 (6.0) |
| **Household member was in prison** |  |  |  |  |
| Yes | n (%) | 20 (1.1) | 10 (1.1) | 10 (1.1) |
| Data are presented as number and (%). | | | | |

| **Supplementary Table 3.** Sex-stratified linear regression analysis of any ACE history and inflammatory biomarkers | | | | | | | | |
| --- | --- | --- | --- | --- | --- | --- | --- | --- |
| **Biomarker** | **Model 1** | | **Model 2** | | **Model 3** | | **Model 4** | |
| ***Males*** | **β (95% CI)** | ***p*** | **β (95% CI)** | ***p*** | **β (95% CI)** | ***p*** | **β (95% CI)** | ***p*** |
| Log CRP | 0.063 (-0.043, 0.169) | 0.242 | 0.091 (-0.013, 0.196) | 0.087 | 0.087 (-0.017, 0.192) | 0.101 | 0.046 (-0.061, 0.143) | 0.397 |
| C3 | -1.049 (-4.416, 2.318) | 0.541 | -1.334 (-4.725, 2.056) | 0.440 | 1.639 (-4.948, 1.670) | 0.331 | -1.553 (-4.890, 1.785) | 0.361 |
| Log IL-6 | 0.025 (-0.093, 0.142) | 0.681 | 0.069 (-0.047, 0.184) | 0.243 | 0.048 (-0.067, 0.162) | 0.413 | 0.011 (-0.106, 0.128) | 0.859 |
| Log TNF-α | 0.053 (-0.001, 0.107) | 0.056 | 0.063 (0.009, 0.117) | **0.022** | 0.060 (0.007, 0.114) | **0.027** | 0.069 (0.012, 0.125) | **0.017** |
| Log leptin | -0.085 (-0.212, 0.042) | 0.191 | -0.091 (-0.219, 0.037) | 0.162 | -0.101 (-0.228, 0.026) | 0.118 | -0.104 (-0.224, 0.017) | 0.092 |
| Log adiponectin | -0.052 (-0.147, 0.044) | 0.288 | -0.034 (-0.129, 0.061) | 0.483 | -0.026 (-0.121, 0.069) | 0.593 | -0.012 (-0.110, 0.085) | 0.806 |
| Log LAR | -0.034 (-0.198, 0.130) | 0.686 | -0.057 (-0.222, 0.107) | 0.495 | -0.076 (-0.239, 0.086) | 0.358 | -0.093 (-0.245, 0.059) | 0.230 |
| Log resistin | 0.058 (-0.008, 0.125) | 0.086 | 0.070 (0.003, 0.136) | **0.04** | 0.062 (-0.005, 0.128) | 0.068 | 0.060 (-0.009, 0.128) | 0.087 |
| GlycA | 0.220 (-9.551, 9.990) | 0.965 | 1.047 (-8.773, 10.867) | 0.834 | 0.052 (-9.285, 10.327) | 0.917 | -2.348 (-12.494, 7.797) | 0.650 |
| Log WBC | 0.011 (-0.032, 0.054) | 0.606 | 0.014 (-0.029, 0.057) | 0.516 | 0.006 (-0.036, 0.048) | 0.778 | -0.015 (-0.056, 0.027) | 0.483 |
| Log monocytes | -0.001 (-0.050, 0.047) | 0.954 | 0.008 (-0.040, 0.056) | 0.74 | -0.001 (-0.049, 0.047) | 0.962 | -0.028 (-0.076, 0.020) | 0.246 |
| Log basophils | 0.040 (-0.047, 0.127) | 0.372 | 0.042 (-0.046, 0.130) | 0.246 | 0.038 (-0.051, 0.126) | 0.404 | 0.034 (-0.057, 0.125) | 0.462 |
| Log eosinophils | 0.006 (-0.090, 0.103) | 0.898 | 0.019 (-0.078, 0.116) | 0.707 | 0.009 (-0.087, 0.106) | 0.848 | -0.004 (-0.105, 0.097) | 0.937 |
| Log neutrophils | 0.007 (-0.004, 0.058) | 0.784 | 0.013 (-0.038, 0.063) | 0.629 | 0.003 (-0.047, 0.053) | 0.914 | -0.026 (-0.075, 0.024) | 0.31 |
| Log lymphocytes | 0.037 (-0.015, 0.089) | 0.164 | 0.031 (-0.022, 0.083) | 0.251 | 0.029 (-0.022, 0.080) | 0.268 | 0.024 (-0.029, 0.076) | 0.372 |
| Log NLR | -0.030 (-0.090, 0.030) | 0.33 | -0.018 (-0.078, 0.042) | 0.555 | -0.026 (-0.086, 0.034) | 0.393 | -0.050 (-0.113, 0.014) | 0.125 |
| PAI-1 | 2.197 (0.282, 4.112) | **0.025** | 2.104 (0.176, 4.031) | **0.032** | 2.146 (0.214, 4.077) | **0.03** | 2.191 (0.213, 4.169) | **0.03** |
| ***Females*** | **β (95% CI)** | ***p*** | **β (95% CI)** | ***p*** | **β (95% CI)** | ***p*** | **β (95% CI)** | ***p*** |
| Log CRP | 0.064 (-0.052, 0.180) | 0.278 | 0.081 (-0.036, 0.198) | 0.172 | 0.084 (-0.032, 0.201) | 0.155 | 0.044 (-0.067, 0.155) | 0.432 |
| C3 | 2.637 (-1.557, 6.831) | 0.218 | 3.522 (-0.713, 7.758) | 0.103 | 3.646 (-0.560, 7.852) | 0.089 | 1.631 (-2.340, 5.602) | 0.42 |
| Log IL-6 | 0.008 (-0.108, 0.124) | 0.897 | 0.065 (-0.050, 0.181) | 0.267 | 0.065 (-0.049, 0.178) | 0.264 | 0.030 (-0.085, 0.144) | 0.611 |
| Log TNF-α | -0.056 (-0.110, -0.001) | **0.045** | -0.032 (-0.086, 0.022) | 0.25 | -0.031 (-0.085, 0.023) | 0.26 | -0.038 (-0.095, 0.019) | 0.193 |
| Log leptin | -0.002 (-0.148, 0.144) | 0.975 | 0.023 (-0.125, 0.171) | 0.762 | 0.022 (-0.125, 0.169) | 0.772 | -0.094 (-0.212, 0.023) | 0.116 |
| Log adiponectin | -0.140 (-0.232, -0.048) | **0.003** | -0.122 (-0.125, -0.029) | **0.01** | -0.124 (-0.215, -0.032) | **0.008** | -0.078 (-0.170, 0.014) | 0.098 |
| Log LAR | 0.137 (-0.052, 0.327) | 0.155 | 0.145 (-0.047, 0.337) | 0.138 | 0.145 (-0.044, 0.334) | 0.132 | -0.017 (-0.168, 0.134) | 0.829 |
| Log resistin | 0.043 (-0.027, 0.113) | 0.231 | 0.039 (-0.032, 0.110) | 0.285 | 0.040 (-0.030, 0.110) | 0.264 | 0.037 (-0.037, 0.110) | 0.328 |
| GlycA | 6.012 (-3.746, 15.771) | 0.227 | 9.143 (-0.645, 18.932) | 0.067 | 9.702 (0.004, 19.401) | 0.05 | 9.389 (-0.530, 19.309) | 0.064 |
| Log WBC | 0.050 (0.007, 0.093) | **0.024** | 0.053 (0.009, 0.096) | **0.018** | 0.053 (0.011, 0.096) | **0.014** | 0.044 (0.001, 0.086) | **0.047** |
| Log monocytes | 0.054 (0.004, 0.104) | **0.034** | 0.064 (0.014, 0.114) | **0.013** | 0.064 (0.014, 0.114) | **0.012** | 0.068 (0.016, 0.120) | **0.01** |
| Log basophils | 0.079 (-0.009, 0.167) | 0.077 | 0.076 (-0.013, 0.165) | 0.095 | 0.076 (-0.014, 0.165) | 0.097 | 0.084 (-0.010, 0.178) | 0.081 |
| Log eosinophils | 0.135 (0.039, 0.230) | **0.006** | 0.133 (0.036, 0.231) | **0.007** | 0.133 (0.036, 0.230) | **0.077** | 0.121 (0.020, 0.221) | **0.019** |
| Log neutrophils | 0.044 (-0.011, 0.099) | 0.120 | 0.049 (-0.007, 0.105) | 0.088 | 0.050 (-0.005, 0.105) | 0.074 | 0.037 (-0.019, 0.093) | 0.193 |
| Log lymphocytes | 0.042 (-0.009, 0.093) | 0.104 | 0.038 (-0.013, 0.089) | 0.147 | 0.038 (-0.014, 0.089) | 0.149 | 0.024 (-0.028, 0.076) | 0.363 |
| Log NLR | 0.002 (-0.060, 0.064) | 0.950 | 0.011 (-0.052, 0.074) | 0.732 | 0.012 (-0.050, 0.075) | 0.704 | 0.013 (-0.053, 0.079) | 0.702 |
| PAI-1 | 0.441 (-1.461, 2.343) | 0.649 | 0.661 (-1.265, 2.587) | 0.501 | 0.671 (-1.257, 2.599) | 0.495 | 0.269 (-1.764, 2.302) | 0.795 |
| Model 1: unadjusted; Model 2: adjusted for age; Model 3: additionally adjusted for education, anti-inflammatory medication use, type 2 diabetes, cardiovascular disease and cancer; Model 4: additionally adjusted for smoking status, alcohol use, diet quality and BMI. Unstandardised β coefficients and 95% confidence intervals (CI) are shown. Significant *p* in **bold**.  C3: complement component 3; CRP: c-reactive protein; GlycA: glycoprotein acetyl; IL-6: interleukin 6; LAR: leptin-adiponectin ratio; NLR: neutrophil-lymphocyte ratio; PAI-1: plasminogen activator inhibitor-1; TNF-a: tumour necrosis factor alpha; WBC: white blood cell count. | | | | | | | | |

| **Supplementary Table 4.** Linear regression analysis of ACE abuse, neglect and household dysfunction exposures and inflammatory biomarkers among male participants | | | | | | | | |
| --- | --- | --- | --- | --- | --- | --- | --- | --- |
| **Biomarker** | **Model 1** | | **Model 2** | | **Model 3** | | **Model 4** | |
| ***Abuse exposure*** | **β (95% CI)** | ***p*** | **β (95% CI)** | ***p*** | **β (95% CI)** | ***p*** | **β (95% CI)** | ***p*** |
| Log CRP | -0.033 (-0.167, 0.102) | 0.631 | -0.014 (-0.148, 0.119) | 0.831 | -0.028 (-0.161, 0.104) | 0.677 | -0.078 (-0.214, 0.057) | 0.256 |
| C3 | -0.854 (-5.137, 3.428) | 0.696 | -1.046 (-5.337, 3.245) | 0.632 | -2.153 (-6.344, 2.038) | 0.314 | -2.700 (-6.934, 1.534) | 0.211 |
| Log IL-6 | -0.002 (-0.152, 0.148) | 0.981 | 0.027 (-0.119, 0.174) | 0.716 | -0.010 (-0.155, 0.135) | 0.893 | -0.033 (-0.182, 0.166) | 0.663 |
| Log TNF-α | 0.051 (-0.018, 0.120) | 0.144 | 0.058 (-0.011, 0.127) | 0.098 | 0.047 (-0.021, 0.115) | 0.176 | 0.056 (-0.016, 0.128) | 0.128 |
| Log leptin | -0.122 (-0.274, 0.050) | 0.174 | -0.116 (-0.278, 0.046) | 0.161 | -0.139 (-0.300, 0.022) | 0.091 | -0.169 (-0.322, -0.106) | **0.031** |
| Log adiponectin | -0.010 (-0.131, 0.112) | 0.876 | 0.002 (-0.119, 0.123) | 0.973 | 0.012 (-0.108, 0.132) | 0.846 | 0.028 (-0.095, 0.152) | 0.652 |
| Log LAR | -0.105 (-0.314, 0.105) | 0.327 | -0.119 (-0.328, 0.089) | 0.262 | -0.153 (-0.359, 0.053) | 0.145 | -0.201 (-0.395, -0.008) | **0.041** |
| Log resistin | 0.094 (0.009, 0.178) | **0.03** | 0.101 (0.017, 0.186) | **0.018** | 0.084 (0.000, 0.168) | **0.049** | 0.069 (-0.018, 0.156) | 0.118 |
| GlycA | -1.155 (-13.595, 11.285) | 0.855 | -0.635 (-13.084, 11.814) | 0.920 | -2.150 (-14.589, 10.289) | 0.735 | -3.913 (-16.815, 8.989) | 0.552 |
| Log WBC | -0.003 (-0.058, 0.052) | 0.92 | -0.001 (-0.056, 0.054) | 0.973 | -0.016 (-0.070, 0.037) | 0.549 | -0.038 (-0.091, 0.015) | 0.163 |
| Log monocytes | -0.029 (-0.091, 0.033) | 0.354 | -0.023 (-0.085, 0.039) | 0.463 | -0.038 (-0.099, 0.023) | 0.223 | -0.061 (-0.123, 0.000) | 0.051 |
| Log basophils | -0.008 (-0.120, 0.104) | 0.893 | -0.006 (-0.119, 0.106) | 0.912 | -0.015 (-0.127, 0.098) | 0.8 | -0.025 (-0.141, 0.091) | 0.67 |
| Log eosinophils | -0.024 (-0.149, 0.100) | 0.699 | -0.016 (-0.140, 0.108) | 0.799 | -0.034 (-0.158, 0.089) | 0.585 | -0.040 (-0.169, 0.090) | 0.548 |
| Log neutrophils | -0.007 (-0.072, 0.058) | 0.835 | -0.003 (-0.068, 0.061) | 0.918 | -0.023 (-0.086, 0.041) | 0.484 | -0.056 (-0.120, 0.007) | 0.082 |
| Log lymphocytes | 0.029 (-0.038, 0.096) | 0.403 | 0.024 (-0.043, 0.091) | 0.477 | 0.011 (-0.055, 0.077) | 0.74 | 0.011 (-0.056, 0.078) | 0.742 |
| Log NLR | -0.035 (-0.113, 0.042) | 0.368 | -0.028 (-0.105, 0.049) | 0.481 | -0.034 (-0.111, 0.043) | 0.389 | -0.067 (-0.148, 0.013) | 0.102 |
| PAI-1 | 1.368 (-1.077, 3.813) | 0.273 | 1.292 (-1.157, 3.741) | 0.301 | 1.197 (-1.259, 3.654) | 0.339 | 1.064 (-1.453, 3.581) | 0.407 |
| ***Neglect exposure*** | **β (95% CI)** | ***p*** | **β (95% CI)** | ***p*** | **β (95% CI)** | ***p*** | **β (95% CI)** | ***p*** |
| Log CRP | 0.018 (-0.177, 0.212) | 0.858 | 0.048 (-0.144, 0.241) | 0.624 | 0.027 (-0.165, 0.220) | 0.781 | -0.068 (-0.274, 0.138) | 0.517 |
| C3 | -7.897 (-14.006, -1.788) | **0.011** | -8.250 (-14.373, -2.127) | **0.008** | -8.087 (-14.086, -2.807) | **0.008** | -9.494 (-15.963, -3.025) | **0.004** |
| Log IL-6 | 0.048 (-0.169, 0.265) | 0.664 | 0.101 (-0.111, 0.313) | 0.349 | 0.100 (-0.110, 0.310) | 0.351 | 0.052 (-0.174, 0.279) | 0.649 |
| Log TNF-α | 0.062 (-0.038, 0.162) | 0.233 | 0.074 (-0.025, 0.174) | 0.144 | 0.070 (-0.029, 0.169) | 0.166 | 0.066 (-0.044, 0.176) | 0.239 |
| Log leptin | -0.144 (-0.375, 0.088) | 0.223 | -0.151 (-0.383, 0.081) | 0.202 | -0.152, -0.384, 0.080) | 0.198 | -0.175 (-0.408, 0.057) | 0.14 |
| Log adiponectin | -0.046 (-0.221, 0.128) | 0.600 | -0.026 (-0.199, 0.148) | 0.772 | -0.023 (-0.196, 0.151) | 0.797 | 0.028 (-0.160, 0.216) | 0.77 |
| Log LAR | -0.096 (-0.396, 0.203) | 0.528 | -0.124 (-0.424, 0.175) | 0.416 | -0.128 (-0.425, 0.168) | 0.395 | -0.202 (-0.494, 0.092) | 0.178 |
| Log resistin | 0.077 (-0.046, 0.199) | 0.22 | 0.090 (-0.032, 0.212) | 0.148 | 0.094 (-0.028, 0.216) | 0.131 | 0.076 (-0.057, 0.208) | 0.264 |
| GlycA | -15.759 (-33.701, 2.182) | 0.085 | -14.876 (-32.850, 3.097) | 0.105 | -14.858 (-32.871, 3.152) | 0.106 | -20.547 (-40.293, -0.802) | **0.041** |
| Log WBC | -0.023 (-0.101, 0.055) | 0.556 | -0.020 (-0.098, 0.058) | 0.617 | -0.020 (-0.097, 0.056) | 0.605 | -.0057 (-0.137, 0.024) | 0.166 |
| Log monocytes | -0.021 (-0.109, 0.067) | 0.642 | -0.009 (-0.097, 0.079) | 0.841 | -0.012 (-0.099, 0.076) | 0.795 | -0.057 (-0.150, 0.036) | 0.227 |
| Log basophils | 0.037 (-0.121, 0.196) | 0.644 | 0.040 (-0.119, 0.199) | 0.623 | 0.040 (-0.120, 0.201) | 0.621 | 0.060 (-0.115, 0.235) | 0.503 |
| Log eosinophils | -0.062 (-0.238, 0.114) | 0.489 | -0.048 (-0.223, 0.128) | 0.595 | -0.050 (-0.226, 0.126) | 0.578 | -0.080 (-0.275, 0.115) | 0.421 |
| Log neutrophils | -0.022 (-0.114, 0.070) | 0.636 | -0.016 (-0.108, 0.077) | 0.74 | -0.009 (-0.099, 0.082) | 0.854 | -0.085 (-0.154, 0.038) | 0.233 |
| Log lymphocytes | -0.026 (-0.121, 0.069) | 0.595 | -0.034 (-0.130, 0.061) | 0.478 | -0.031 (-0.125, 0.062) | 0.513 | -0.035 (-0.136, 0.066) | 0.497 |
| Log NLR | 0.004 (-0.106, 0.114) | 0.95 | 0.019 (-0.091, 0.128) | 0.736 | 0.023 (-0.087, 0.133) | 0.685 | -0.023 (-0.146, 0.099) | 0.709 |
| PAI-1 | 2.755 (-0.776. 6.285) | 0.126 | 2.623 (0.918, 6.164) | 0.146 | 2.692 (-0.869, 6.254) | 0.138 | 2.183 (-1.653, 6.018) | 0.264 |
| ***Household dysfunction exposure*** | **β (95% CI)** | ***p*** | **β (95% CI)** | ***p*** | **β (95% CI)** | ***p*** | **β (95% CI)** | ***p*** |
| Log CRP | 0.126 (0.003, 0.250) | **0.045** | 0.167 (0.044, 0.290) | **0.008** | 0.169 (0.047, 0.291) | **0.007** | 0.106 (-0.018, 0.231) | 0.095 |
| C3 | 1.802 (-2.144, 5.718) | 0.367 | 1.478 (-2.475, 5.431) | 0.463 | 1.388 (-2.469, 5.245) | 0.48 | 1.371 (-2.523, 5.264) | 0.49 |
| Log IL-6 | 0.063 (-0.075, 0.201) | 0.369 | 0.126 (-0.010, 0.262) | 0.069 | 0.111 (-0.023, 0.245) | 0.106 | 0.046 (-0.091, 0.183) | 0.511 |
| Log TNF-α | 0.048 (-0.015, 0.112) | 0.136 | 0.063 (-0.001, 0.127) | 0.053 | 0.064 (0.001, 0.127) | **0.047** | 0.064 (-0.003, 0.130) | 0.06 |
| Log leptin | 0.020 (-0.128, 0.168) | 0.792 | 0.013 (-0.137, 0.162) | 0.869 | 0.008 (-0.141, 0.156) | 0.921 | -0.030 (-0.171, 0.111) | 0.675 |
| Log adiponectin | -0.060 (-0.172, 0.051) | 0.289 | -0.036 (-0.148, 0.076) | 0.528 | -0.027 (-0.139, 0.084) | 0.631 | -0.031 (-0.144, 0.083) | 0.599 |
| Log LAR | 0.081 (-0.111, 0.273) | 0.407 | 0.050 (-0.144, 0.243) | 0.614 | 0.036 (-0.155, 0.226) | 0.713 | 0.002 (-0.176, 0.180) | 0.984 |
| Log resistin | 0.024 (-0.054, 0.102) | 0.552 | 0.040 (-0.039, 0.118) | 0.323 | 0.034 (-0.044, 0.112) | 0.394 | 0.018 (-0.062, 0.099) | 0.652 |
| GlycA | 9.026 (-2.406, 20.457) | 0.122 | 10.347 (-1.168, 21.862) | 0.078 | 10.139 (-1.348, 21.626) | 0.084 | 5.758 (-6.126, 17.642) | 0.342 |
| Log WBC | 0.040 (-0.010, 0.090) | 0.120 | 0.044 (-0.006, 0.095) | 0.083 | 0.038 (0.011, 0.087) | 0.131 | 0.012 (-0.037, 0.061) | 0.627 |
| Log monocytes | 0.020 (-0.037, 0.076) | 0.493 | 0.034 (-0.023, 0.091) | 0.238 | 0.026 (-0.030, 0.082) | 0.356 | -0.006 (-0.062, 0.050) | 0.837 |
| Log basophils | 0.090 (-0.011, 0.192) | 0.082 | 0.094 (-0.008, 0.197) | 0.071 | 0.091 (-0.012 (0.194) | 0.084 | 0.058 (-0.048, 0.164) | 0.282 |
| Log eosinophils | 0.012 (-0.101, 0.125) | 0.83 | 0.030 (-0.084, 0.143) | 0.609 | 0.023 (-0.091, 0.136) | 0.695 | 0.005 (-0.113, 0.123) | 0.93 |
| Log neutrophils | 0.042 (-0.017, 0.101) | 0.164 | 0.050 (-0.009, 0.110) | 0.096 | 0.042 (-0.016, 0.100) | 0.158 | 0.008 (-0.050, 0.066) | 0.781 |
| Log lymphocytes | 0.057 (-0.004, 0.118) | 0.066 | 0.048 (-0.013, 0.110) | 0.123 | 0.049 (-0.011, 0.110) | 0.107 | 0.043 (-0.018, 0.104) | 0.169 |
| Log NLR | -0.015 (-0.086, 0.055) | 0.670 | 0.002 (-0.069, 0.073) | 0.953 | -0.007 (-0.078, 0.063) | 0.836 | -0.035 (-0.109, 0.039) | 0.358 |
| PAI-1 | 3.154 (0.913, 5.395) | **0.006** | 3.041 (0.778, 5.303) | **0.008** | 3.113 (0.848, 5.377) | **0.007** | 2.630 (0.314, 4.945) | **0.026** |
| Model 1: unadjusted; Model 2: adjusted for age; Model 3: additionally adjusted for education, anti-inflammatory medication use, type 2 diabetes, cardiovascular disease and cancer; Model 4: additionally adjusted for smoking status, alcohol use, diet quality and BMI. Unstandardised β coefficients and 95% confidence intervals (CI) are shown. Significant *p* in **bold**.  C3: complement component 3; CRP: c-reactive protein; GlycA: glycoprotein acetyl; IL-6: interleukin 6; LAR: leptin-adiponectin ratio; NLR: neutrophil-lymphocyte ratio; PAI-1: plasminogen activator inhibitor-1; TNF-a: tumour necrosis factor alpha; WBC: white blood cell count. | | | | | | | | |

| **Supplementary Table 5.** Linear regression analysis of ACE abuse, neglect and household dysfunction exposures and inflammatory biomarkers among female participants | | | | | | | | |
| --- | --- | --- | --- | --- | --- | --- | --- | --- |
| **Biomarker** | **Model 1** | | **Model 2** | | **Model 3** | | **Model 4** | |
| ***Abuse exposure*** | **β (95% CI)** | ***p*** | **β (95% CI)** | ***p*** | **β (95% CI)** | ***p*** | **β (95% CI)** | ***p*** |
| Log CRP | 0.042 (-0.016, 0.190) | 0.579 | 0.063 (-0.087, 0.213) | 0.408 | 0.054 (-0.095, 0.203) | 0.474 | 0.016 (-0.125, 0.157) | 0.825 |
| C3 | 3.538 (-1.826, 8.903) | 0.196 | 4.675 (-0.743, 10.092) | 0.091 | 4.466 (-0.910, 9.843) | 0.103 | 2.494 (-2.545, 7.533) | 0.332 |
| Log IL-6 | 0.021 (-0.127, 0.170) | 0.777 | 0.095 (-0.052, 0.243) | 0.205 | 0.084 (-0.061, 0.229) | 0.256 | 0.056 (-0.089, 0.201) | 0.448 |
| Log TNF-α | -0.037 (-0.107, 0.032) | 0.292 | -0.006 (-0.076, 0.063) | 0.860 | -0.006 (-0.075, 0.063) | 0.868 | -0.011 (-0.084, 0.062) | 0.769 |
| Log leptin | 0.045 (-0.142, 0.232) | 0.637 | 0.078 (-0.011, 0.267) | 0.416 | 0.074 (-0.114, 0.262) | 0.44 | -0.029 (-0.178, 0.120) | 0.702 |
| Log adiponectin | -0.147 (-0.265, -0.029) | **0.015** | -0.124 (-0.243, -0.004) | **0.042** | -0.123 (-0.240, -0.007) | **0.039** | -0.087 (-0.203, 0.030) | 0.146 |
| Log LAR | 0.192 (-0.050, 0.434) | 0.120 | 0.202 (-0.043, 0.448) | 0.106 | 0.197 (-0.044, 0.439) | 0.109 | 0.057 (-0.134, 0.249) | 0.556 |
| Log resistin | 0.097 (0.007, 0.186) | **0.034** | 0.093 (0.002, 0.183) | **0.045** | 0.087 (-0.002, 0.177) | 0.056 | 0.076 (-0.017, 0.170) | 0.11 |
| GlycA | 7.197 (-5.287, 19.680) | 0.258 | 11.261 (-1.266, 23.788) | 0.078 | 10.868 (-1.537, 23.272) | 0.086 | 8.675 (-3.938, 21.287) | 0.177 |
| Log WBC | 0.082 (0.026, 0.137) | **0.004** | 0.086 (0.030, 0.142) | **0.003** | 0.084 (0.029, 0.138) | **0.003** | 0.067 (0.012, 0.122) | **0.016** |
| Log monocytes | 0.087 (0.023, 0.151) | **0.008** | 0.100 (0.035, 0.165) | **0.003** | 0.098 (0.033, 0.162) | **0.003** | 0.096 (0.030, 0.162) | **0.005** |
| Log basophils | 0.113 (0.000, 0.227) | **0.049** | 0.109 (-0.005, 0.224) | 0.061 | 0.108 (-0.006, 0.223) | 0.064 | 0.109 (-0.011, 0.229) | 0.075 |
| Log eosinophils | 0.157 (0.034, 0.281) | **0.013** | 0.155 (0.030, 0.280) | **0.015** | 0.153 (0.029, 0.278) | **0.016** | 0.120 (-0.008, 0.249) | 0.066 |
| Log neutrophils | 0.060 (-0.012, 0.131) | 0.101 | 0.066 (-0.006, 0.138) | 0.073 | 0.062 (-0.009, 0.133) | 0.085 | 0.040 (-0.032, 0.111) | 0.277 |
| Log lymphocytes | 0.114 (0.049, 0.178) | **0.001** | 0.110 (0.044, 0.175) | **0.001** | 0.111 (0.045, 0.176) | **0.001** | **0.099** (0.033, 0.166) | **0.003** |
| Log NLR | -0.054 (-0.134, 0.026) | 0.188 | -0.044 (-0.125, 0.037) | 0.290 | -0.049 (-0.129, 0.032) | 0.236 | -0.060 (-0.144, 0.025) | 0.166 |
| PAI-1 | 0.962 (-1.471, 3.395) | 0.438 | 1.254 (-1.210, 3.717) | 0.318 | 1.174 (-1.290, 3.638) | 0.35 | 0.561 (-2.020, 3.142) | 0.67 |
| ***Neglect exposure*** | **β (95% CI)** | ***p*** | **β (95% CI)** | ***p*** | **β (95% CI)** | ***p*** | **β (95% CI)** | ***p*** |
| Log CRP | 0.108 (-0.075, 0.291) | 0.248 | 0.125 (-0.059, 0.309) | 0.182 | 0.109 (-0.074, 0.292) | 0.243 | 0.006 (-0.171, 0.182) | 0.95 |
| C3 | 9.549 (2.936, 16.162) | **0.005** | 10.496 (3.868, 17.124) | **0.002** | 9.781 (3.196, 16.366) | **0.004** | 5.616 (-0.695, 11.927) | 0.081 |
| Log IL-6 | 0.201 (0.018, 0.385) | **0.031** | 0.263 (0.082, 0.443) | **0.004** | 0.232 (0.054, 0.410) | **0.011** | 0.118 (-0.064, 0.300) | 0.203 |
| Log TNF-α | -0.016 (-0.103, 0.070) | 0.709 | 0.009 (-0.076, 0.095) | 0.830 | 0.002 (-0.084, 0.087) | 0.971 | -0.012 (-0.104, 0.079) | 0.791 |
| Log leptin | 0.228 (-0.003, 0.459) | 0.053 | 0.256 (0.025, 0.488) | **0.03** | 0.231 (0.001, 0.462) | **0.049** | -0.037 (-0.224, 0.151) | 0.701 |
| Log adiponectin | -0.136 (-0.283, 0.010) | 0.068 | -0.116 (-0.263, 0.031) | 0.121 | -0.096 (-0.239, 0.048) | 0.192 | -0.015 (-0.162, 0.132) | 0.842 |
| Log LAR | 0.364 (0.065, 0.663) | **0.017** | 0.372 (0.071, 0.673) | **0.015** | 0.327 (0.030, 0.624) | **0.031** | -0.022 (-0.262, 0.219) | 0.859 |
| Log resistin | 0.003 (-0.108, 0.114) | 0.957 | -0.002 (-0.114, 0.109) | 0.969 | -0.019 (-0.129, 0.091) | 0.734 | -0.046 (-0.164, 0.071) | 0.438 |
| GlycA | 11.546 (-3.931, 27.023) | 0.144 | 14.736 (-0.689, 30.161) | 0.061 | 13.263 (-2.025, 28.551) | 0.089 | 10.703 (-5.163, 26.659) | 0.186 |
| Log WBC | 0.048 (-0.021, 0.116) | 0.171 | 0.050 (-0.019, 0.118) | 0.152 | 0.038 (-0.030, 0.105) | 0.273 | 0.002 (-0.067, 0.070) | 0.959 |
| Log monocytes | 0.071 (-0.008, 0.150) | 0.079 | 0.081 (0.002, 0.160) | **0.045** | 0.071 (-0.008, 0.150) | 0.077 | 0.068 (-0.015, 0.151) | 0.111 |
| Log basophils | 0.062 (-0.077, 0.201) | 0.380 | 0.057 (-0.083, 0.197) | 0.423 | 0.051 (-0.089, 0.192) | 0.474 | 0.048 (-0.102, 0.198) | 0.53 |
| Log eosinophils | 0.183 (0.031, 0.336) | **0.018** | 0.181 (0.028, 0.334) | **0.020** | 0.170 (0.018, 0.323) | **0.029** | 0.099 (-0.062, 0.260) | 0.227 |
| Log neutrophils | 0.045 (-0.043, 0.132) | 0.316 | 0.049 (-0.039, 0.137) | 0.271 | 0.032 (-0.054, 0.118) | 0.463 | -0.009 (-0.098, 0.080) | 0.84 |
| Log lymphocytes | 0.035 (-0.045, 0.115) | 0.395 | 0.030 (-0.051, 0.110) | 0.470 | 0.026 (-0.055, 0.107) | 0.529 | -0.014 (-0.097, 0.069) | 0.734 |
| Log NLR | 0.010 (-0.089, 0.109) | 0.841 | 0.020 (-0.079, 0.119) | 0.695 | 0.006 (-0.092, 0.105) | 0.899 | 0.005 (-0.100, 0.110) | 0.923 |
| PAI-1 | -0.457 (-3.468, 2.554) | 0.766 | -0.247 (-3.274, 2.779) | 0.873 | -0.441 (-3.470, 2.588) | 0.775 | -1.143 (-4.379, 2.093) | 0.488 |
| ***Household dysfunction exposure*** | **β (95% CI)** | ***p*** | **β (95% CI)** | ***p*** | **β (95% CI)** | ***p*** | **β (95% CI)** | ***p*** |
| Log CRP | 0.050 (-0.088, 0.188) | 0.478 | 0.070 (-0.069, 0.210) | 0.324 | 0.074 (-0.065, 0.214) | 0.294 | 0.045 (-0.086, 0.177) | 0.5 |
| C3 | 0.491 (-4.513, 5.495) | 0.847 | 1.486 (-3.571, 6.543) | 0.564 | 1.695 (-3.344, 6.733) | 0.509 | -0.195 (-4.898, 4.508) | 0.935 |
| Log IL-6 | 0.068 (-0.071, 0.206) | 0.338 | 0.138 (0.001, 0.276) | **0.048** | 0.139 (0.003, 0.274) | **0.045** | 0.139 (0.004, 0.274) | **0.043** |
| Log TNF-α | -0.032 (-0.097, 0.033) | 0.332 | -0.003 (-0.068, 0.062) | 0.934 | -0.002 (-0.067, 0.063) | 0.956 | -0.004 (-0.072, 0.064) | 0.902 |
| Log leptin | -0.087 (-0.261, 0.087) | 0.329 | -0.059 (-0.235, 0.117) | 0.513 | -0.060 (-0.236, 0.116) | 0.502 | -0.140 (-0.279, -0.001) | **0.048** |
| Log adiponectin | -0.149 (-0.259, -0.039) | **0.008** | -0.128 (-0.239, -0.017) | **0.024** | -0.130 (-0.239, -0.021) | **0.02** | -0.071 (-0.180, 0.038) | 0.204 |
| Log LAR | 0.063 (-0.163, 0.289) | 0.586 | 0.069 (-0.160, 0.298) | 0.554 | 0.070 (-0.157, 0.296) | 0.547 | -0.070 (-0.248, 0.109) | 0.443 |
| Log resistin | 0.044 (-0.040, 0.127) | 0.306 | 0.039 (-0.046, 0.123) | 0.372 | 0.042 (-0.042, 0.126) | 0.324 | 0.055 (-0.033, 0.142) | 0.218 |
| GlycA | 3.136 (-8.496, 14.769) | 0.597 | 6.896 (-4.787, 18.579) | 0.247 | 7.789 (-3.834, 19.401) | 0.188 | 7.149 (-4.602, 18.901) | 0.233 |
| Log WBC | 0.046 (-0.006, 0.097) | 0.081 | 0.049 (-0.003, 0.101) | 0.065 | 0.050 (-0.001, 0.101) | 0.056 | 0.041 (-0.010, 0.092) | 0.115 |
| Log monocytes | 0.059 (0.000, 0.119) | 0.050 | 0.072 (0.012, 0.132) | **0.019** | 0.071 (0.011, 0.131) | **0.02** | 0.067 (0.006, 0.129) | **0.032** |
| Log basophils | 0.108 (0.003, 0.212) | **0.044** | 0.104 (-0.002, 0.210) | 0.055 | 0.104 (-0.003, 0.211) | 0.056 | 0.117 (0.006, 0.228) | **0.039** |
| Log eosinophils | 0.152 (0.038, 0.266) | **0.009** | 0.151 (0.035, 0.266) | **0.011** | 0.153 (0.037, 0.268) | **0.01** | 0.168 (0.050, 0.287) | **0.006** |
| Log neutrophils | 0.058 (-0.008, 0.124) | 0.083 | 0.065 (-0.002, 0.131) | 0.058 | 0.066 (0.000, 0.131) | **0.049** | 0.054 (-0.012, 0.120) | 0.108 |
| Log lymphocytes | -0.008 (-0.068, 0.053) | 0.806 | -0.014 (-0.075, 0.047) | 0.654 | -0.015 (-0.077, 0.046) | 0.628 | -0.027 (-0.089, 0.035) | 0.388 |
| Log NLR | 0.066 (-0.008, 0.140) | 0.082 | 0.078 (0.003, 0.153) | **0.04** | 0.081 (0.006, 0.156) | **0.033** | 0.081 (0.003, 0.159) | **0.041** |
| PAI-1 | -0.843 (-3.110, 1.425) | 0.466 | -0.614 (-2.912, 1.683) | 0.600 | -0.620 (-2.927, 1.687) | 0.598 | -1.216 (-3.622, 1.190) | 0.321 |
| Model 1: unadjusted; Model 2: adjusted for age; Model 3: additionally adjusted for education, anti-inflammatory medication use, type 2 diabetes, cardiovascular disease and cancer; Model 4: additionally adjusted for smoking status, alcohol use, diet quality and BMI. Unstandardised β coefficients and 95% confidence intervals (CI) are shown. Significant *p* in **bold**.  C3: complement component 3; CRP: c-reactive protein; GlycA: glycoprotein acetyl; IL-6: interleukin 6; LAR: leptin-adiponectin ratio; NLR: neutrophil-lymphocyte ratio; PAI-1: plasminogen activator inhibitor-1; TNF-a: tumour necrosis factor alpha; WBC: white blood cell count. | | | | | | | | |

**Supplementary information: false discovery rate (FDR) adjusted *p* values**

| Linear regression analysis of any ACE history and inflammatory biomarkers – full sample | | | |
| --- | --- | --- | --- |
| **Biomarker** | **Model 4** | | |
|  | **β (95% CI)** | ***p*** | ***p* (FDR)** |
| Log CRP | 0.047 (-0.030, 0.124) | 0.232 | 0.899 |
| C3 | -0.047 (-2.640, 2.546) | 0.972 | 0.983 |
| Log IL-6 | 0.020 (-0.062, 0.101) | 0.636 | 0.95 |
| Log TNF-α | 0.018 (-0.023, 0.058) | 0.387 | 0.948 |
| Log leptin | -0.097 (-0.182, -0.013) | **0.024** | 0.3 |
| Log adiponectin | -0.045 (-0.112, 0.022) | 0.186 | 0.866 |
| LAR | -0.053 (-0.161, 0.054) | 0.332 | 0.942 |
| Log resistin | 0.050 (-0.001, 0.100) | 0.053 | 0.532 |
| GlycA | 3.028 (-4.092, 10.148) | 0.404 | 0.948 |
| Log WBC | 0.013 (-0.016, 0.043) | 0.375 | 0.946 |
| Log monocytes | 0.020 (-0.016, 0.055) | 0.277 | 0.916 |
| Log basophils | 0.056 (-0.009, 0.122) | 0.09 | 0.727 |
| Log eosinophils | 0.058 (-0.014, 0.129) | 0.112 | 0.733 |
| Log neutrophils | 0.004 (-0.033, 0.041) | 0.829 | 0.971 |
| Log lymphocytes | 0.024 (-0.013, 0.061) | 0.199 | 0.867 |
| Log NLR | -0.020 (-0.066, 0.026) | 0.39 | 0.948 |
| PAI-1 | 1.223 (-0.191, 2.637) | 0.09 | 0.727 |
| Model 4: adjusted for age, sex, education, anti-inflammatory medication use, type 2 diabetes, cardiovascular disease, cancer, smoking status, alcohol use, diet quality and BMI. Unstandardised β coefficients and 95% confidence intervals (CI) are shown.  Significant *p* in **bold**. | | | |

| Linear regression analysis of ACE abuse, neglect and household dysfunction exposures and inflammatory biomarkers – full sample | | | |
| --- | --- | --- | --- |
| **Biomarker** | **Model 4** |  |  |
| ***Abuse exposure*** | **β (95% CI)** | ***p*** | ***p* (FDR)** |
| Log CRP | -0.027 (-0.125, 0.071) | 0.585 | 0.995 |
| C3 | -0.167 (-3.454, 3.120) | 0.921 | 0.995 |
| Log IL-6 | 0.013 (-0.091, 0.116) | 0.811 | 0.995 |
| Log TNF-α | 0.024 (-0.027, 0.075) | 0.361 | 0.994 |
| Log leptin | -0.103 (-0.210, 0.004) | 0.06 | 0.52 |
| Log adiponectin | -0.027 (-0.112, 0.058) | 0.534 | 0.995 |
| Log LAR | -0.078 (-0.215, 0.058) | 0.261 | 0.974 |
| Log resistin | 0.076 (0.012, 0.139) | **0.02** | 0.265 |
| GlycA | 1.831 (-7.205, 10.687 | 0.691 | 0.995 |
| Log WBC | 0.013 (-0.025, 0.051) | 0.499 | 0.995 |
| Log monocytes | 0.016 (-0.029, 0.061) | 0.491 | 0.995 |
| Log basophils | 0.038 (-0.045, 0.122) | 0.368 | 0.994 |
| Log eosinophils | 0.039 (-0.052, 0.130) | 0.396 | 0.994 |
| Log neutrophils | -0.008 (-0.056, 0.039) | 0.73 | 0.995 |
| Log lymphocytes | 0.054 (0.007, 0.101) | **0.025** | 0.295 |
| Log NLR | -0.062 (-0.121, -0.004) | **0.037** | 0.39 |
| PAI-1 | 0.772 (-1.022, 2.565) | 0.399 | 0.994 |
| ***Neglect exposure*** | **β (95% CI)** | ***p*** | ***p* (FDR)** |
| Log CRP | -0.021 (-0.155, 0.113) | 0.757 | 1.0 |
| C3 | -0.844 (-5.354, 3.666) | 0.714 | 1.0 |
| Log IL-6 | 0.088 (-0.054, 0.229) | 0.224 | 0.965 |
| Log TNF-α | 0.021 (-0.049, 0.090) | 0.563 | 1.0 |
| Log leptin | -0.078 (-0.224, 0.069) | 0.297 | 0.993 |
| Log adiponectin | -0.015 (-0.131, 0.102) | 0.805 | 1.0 |
| Log LAR | **-**0.063 (-0.249, 0.124) | 0.509 | 1.0 |
| Log resistin | 0.004 (-0.084, 0.091) | 0.932 | 1.0 |
| GlycA | -2.776 (-15.209, 9.657) | 0.662 | 1.0 |
| Log WBC | -0.022 (-0.073, 0.030) | 0.413 | 0.998 |
| Log monocytes | 0.015 (-0.046, 0.076) | 0.634 | 1.0 |
| Log basophils | 0.040 (-0.073, 0.153) | 0.488 | 1.0 |
| Log eosinophils | 0.28 (-0.095, 0.152) | 0.651 | 1.0 |
| Log neutrophils | -0.030 (-0.095, 0.035) | 0.365 | 0.996 |
| Log lymphocytes | -0.028 (-0.092, 0.036) | 0.393 | 0.998 |
| Log NLR | -0.002 (-0.082, 0.087) | 0.96 | 1.0 |
| PAI-1 | 0.119 (-2.338, 2.475) | 0.925 | 1.0 |
| ***Household dysfunction exposure*** | **β (95% CI)** | ***p*** | ***p* (FDR)** |
| Log CRP | 0.078 (-0.013, 0.169) | .092 | 0.695 |
| C3 | 0.574 (-2.473, 3.622) | 0.712 | 0.943 |
| Log IL-6 | 0.090 (-0.006, 0.186) | 0.066 | 0.599 |
| Log TNF-α | 0.031 (-0.016, 0.079) | 0.197 | 0.823 |
| Log leptin | -0.082 (-0.182, 0.017) | 0.104 | 0.716 |
| Log adiponectin | -0.049 (-0.128, 0.030) | 0.222 | 0.824 |
| Log LAR | -0.033 (-0.159, 0.094) | 0.612 | 0.943 |
| Log resistin | 0.037 (-0.022, 0.096) | 0.221 | 0.824 |
| GlycA | 6.234 (-2.149, 14.617) | 0.145 | 0.811 |
| Log WBC | 0.026 (-0.009, 0.061) | 0.143 | 0.806 |
| Log monocytes | 0.030 (-0.012, 0.071) | 0.159 | 0.811 |
| Log basophils | 0.087 (0.011, 0.164) | **0.026** | 0.345 |
| Log eosinophils | 0.084 (0.000, 0.167) | 0.05 | 0.532 |
| Log neutrophils | 0.030 (-0.014, 0.074) | 0.181 | 0.819 |
| Log lymphocytes | 0.011 (-0.033, 0.054) | 0.632 | 0.943 |
| Log NLR | 0.019 (-0.035, 0.073) | 0.482 | 0.922 |
| PAI-1 | 0.795 (-0.872, 2.461) | 0.35 | 0.891 |
| Model 4: adjusted for age, sex, education, anti-inflammatory medication use, type 2 diabetes, cardiovascular disease, cancer, smoking status, alcohol use, diet quality and BMI. Unstandardised β coefficients and 95% confidence intervals (CI) are shown.  Significant *p* in **bold**. | | | |

| Sex-stratified linear regression analysis of any ACE history and inflammatory biomarkers | | | |
| --- | --- | --- | --- |
| **Biomarker** | **Model 4** | |  |
| ***Males*** | **β (95% CI)** | ***p*** | ***p* (FDR)** |
| Log CRP | 0.046 (-0.061, 0.143) | 0.397 | 0.98 |
| C3 | -1.553 (-4.890, 1.785) | 0.361 | 0.98 |
| Log IL-6 | 0.011 (-0.106, 0.128) | 0.859 | 0.993 |
| Log TNF-α | 0.069 (0.012, 0.125) | **0.017** | 0.274 |
| Log leptin | -0.104 (-0.224, 0.017) | 0.092 | 0.694 |
| Log adiponectin | -0.012 (-0.110, 0.085) | 0.806 | 0.993 |
| Log LAR | -0.093 (-0.245, 0.059) | 0.230 | 0.927 |
| Log resistin | 0.060 (-0.009, 0.128) | 0.087 | 0.694 |
| GlycA | -2.348 (-12.494, 7.797) | 0.650 | 0.98 |
| Log WBC | -0.015 (-0.056, 0.027) | 0.483 | 0.98 |
| Log monocytes | -0.028 (-0.076, 0.020) | 0.246 | 0.933 |
| Log basophils | 0.034 (-0.057, 0.125) | 0.462 | 0.98 |
| Log eosinophils | -0.004 (-0.105, 0.097) | 0.937 | 0.993 |
| Log neutrophils | -0.026 (-0.075, 0.024) | 0.31 | 0.964 |
| Log lymphocytes | 0.024 (-0.029, 0.076) | 0.372 | 0.98 |
| Log NLR | -0.050 (-0.113, 0.014) | 0.125 | 0.766 |
| PAI-1 | 2.191 (0.213, 4.169) | **0.03** | 0.396 |
| ***Females*** | **β (95% CI)** | ***p*** | ***p* (FDR)** |
| Log CRP | 0.044 (-0.067, 0.155) | 0.432 | 0.967 |
| C3 | 1.631 (-2.340, 5.602) | 0.42 | 0.967 |
| Log IL-6 | 0.030 (-0.085, 0.144) | 0.611 | 0.984 |
| Log TNF-α | -0.038 (-0.095, 0.019) | 0.193 | 0.85 |
| Log leptin | -0.094 (-0.212, 0.023) | 0.116 | 0.695 |
| Log adiponectin | -0.078 (-0.170, 0.014) | 0.098 | 0.664 |
| Log LAR | -0.017 (-0.168, 0.134) | 0.829 | 0.984 |
| Log resistin | 0.037 (-0.037, 0.110) | 0.328 | 0.955 |
| GlycA | 9.389 (-0.530, 19.309) | 0.064 | 0.554 |
| Log WBC | 0.044 (0.001, 0.086) | **0.047** | 0.45 |
| Log monocytes | 0.068 (0.016, 0.120) | **0.01** | 0.136 |
| Log basophils | 0.084 (-0.010, 0.178) | 0.081 | 0.618 |
| Log eosinophils | 0.121 (0.020, 0.221) | **0.019** | 0.223 |
| Log neutrophils | 0.037 (-0.019, 0.093) | 0.193 | 0.85 |
| Log lymphocytes | 0.024 (-0.028, 0.076) | 0.363 | 0.956 |
| Log NLR | 0.013 (-0.053, 0.079) | 0.702 | 0.984 |
| PAI-1 | 0.269 (-1.764, 2.302) | 0.795 | 0.984 |
| Model 4: adjusted for age, education, anti-inflammatory medication use, type 2 diabetes, cardiovascular disease, cancer, smoking status, alcohol use, diet quality and BMI. Unstandardised β coefficients and 95% confidence intervals (CI) are shown.  Significant *p* in **bold**. | | | |

| Linear regression analysis of ACE abuse, neglect and household dysfunction exposures and inflammatory biomarkers among male participants | | | |
| --- | --- | --- | --- |
| **Biomarker** | **Model 4** | |  |
| ***Abuse exposure*** | **β (95% CI)** | ***p*** | ***p* (FDR)** |
| Log CRP | -0.078 (-0.214, 0.057) | 0.256 | 0.876 |
| C3 | -2.700 (-6.934, 1.534) | 0.211 | 0.848 |
| Log IL-6 | -0.033 (-0.182, 0.166) | 0.663 | 0.988 |
| Log TNF-α | 0.056 (-0.016, 0.128) | 0.128 | 0.753 |
| Log leptin | -0.169 (-0.322, -0.106) | **0.031** | 0.391 |
| Log adiponectin | 0.028 (-0.095, 0.152) | 0.652 | 0.988 |
| Log LAR | -0.201 (-0.395, -0.008) | **0.041** | 0.456 |
| Log resistin | 0.069 (-0.018, 0.156) | 0.118 | 0.753 |
| GlycA | -3.913 (-16.815, 8.989) | 0.552 | 0.988 |
| Log WBC | -0.038 (-0.091, 0.015) | 0.163 | 0.775 |
| Log monocytes | -0.061 (-0.123, 0.000) | 0.051 | 0.489 |
| Log basophils | -0.025 (-0.141, 0.091) | 0.67 | 0.988 |
| Log eosinophils | -0.040 (-0.169, 0.090) | 0.548 | 0.988 |
| Log neutrophils | -0.056 (-0.120, 0.007) | 0.082 | 0.657 |
| Log lymphocytes | 0.011 (-0.056, 0.078) | 0.742 | 0.988 |
| Log NLR | -0.067 (-0.148, 0.013) | 0.102 | 0.718 |
| PAI-1 | 1.064 (-1.453, 3.581) | 0.407 | 0.97 |
| ***Neglect exposure*** | **β (95% CI)** | ***p*** | ***p* (FDR)** |
| Log CRP | -0.068 (-0.274, 0.138) | 0.517 | 0.977 |
| C3 | -9.494 (-15.963, -3.025) | **0.004** | 0.091 |
| Log IL-6 | 0.052 (-0.174, 0.279) | 0.649 | 0.977 |
| Log TNF-α | 0.066 (-0.044, 0.176) | 0.239 | 0.931 |
| Log leptin | -0.175 (-0.408, 0.057) | 0.14 | 0.83 |
| Log adiponectin | 0.028 (-0.160, 0.216) | 0.77 | 0.977 |
| Log LAR | -0.202 (-0.494, 0.092) | 0.178 | 0.897 |
| Log resistin | 0.076 (-0.057, 0.208) | 0.264 | 0.931 |
| GlycA | -20.547 (-40.293, -0.802) | **0.041** | 0.454 |
| Log WBC | -.0057 (-0.137, 0.024) | 0.166 | 0.882 |
| Log monocytes | -0.057 (-0.150, 0.036) | 0.227 | 0.931 |
| Log basophils | 0.060 (-0.115, 0.235) | 0.503 | 0.977 |
| Log eosinophils | -0.080 (-0.275, 0.115) | 0.421 | 0.977 |
| Log neutrophils | -0.085 (-0.154, 0.038) | 0.233 | 0.931 |
| Log lymphocytes | -0.035 (-0.136, 0.066) | 0.497 | 0.977 |
| Log NLR | -0.023 (-0.146, 0.099) | 0.709 | 0.977 |
| PAI-1 | 2.183 (-1.653, 6.018) | 0.264 | 0.931 |
| ***Household dysfunction exposure*** | **β (95% CI)** | ***p*** | ***p* (FDR)** |
| Log CRP | 0.106 (-0.018, 0.231) | 0.095 | 0.728 |
| C3 | 1.371 (-2.523, 5.264) | 0.49 | 0.994 |
| Log IL-6 | 0.046 (-0.091, 0.183) | 0.511 | 0.995 |
| Log TNF-α | 0.064 (-0.003, 0.130) | 0.06 | 0.59 |
| Log leptin | -0.030 (-0.171, 0.111) | 0.675 | 0.998 |
| Log adiponectin | -0.031 (-0.144, 0.083) | 0.599 | 0.995 |
| Log LAR | 0.002 (-0.176, 0.180) | 0.984 | 0.998 |
| Log resistin | 0.018 (-0.062, 0.099) | 0.652 | 0.998 |
| GlycA | 5.758 (-6.126, 17.642) | 0.342 | 0.984 |
| Log WBC | 0.012 (-0.037, 0.061) | 0.627 | 0.997 |
| Log monocytes | -0.006 (-0.062, 0.050) | 0.837 | 0.998 |
| Log basophils | 0.058 (-0.048, 0.164) | 0.282 | 0.973 |
| Log eosinophils | 0.005 (-0.113, 0.123) | 0.93 | 0.998 |
| Log neutrophils | 0.008 (-0.050, 0.066) | 0.781 | 0.998 |
| Log lymphocytes | 0.043 (-0.018, 0.104) | 0.169 | 0.876 |
| Log NLR | -0.035 (-0.109, 0.039) | 0.358 | 0.984 |
| PAI-1 | 2.630 (0.314, 4.945) | **0.026** | 0.382 |
| Model 4: adjusted for age, education, anti-inflammatory medication use, type 2 diabetes, cardiovascular disease, cancer, smoking status, alcohol use, diet quality and BMI. Unstandardised β coefficients and 95% confidence intervals (CI) are shown.  Significant *p* in **bold**. | | | |

| Linear regression analysis of ACE abuse, neglect and household dysfunction exposures and inflammatory biomarkers among female participants | | | |
| --- | --- | --- | --- |
| **Biomarker** | **Model 4** | |  |
| ***Abuse exposure*** | **β (95% CI)** | ***p*** | ***p* (FDR)** |
| Log CRP | 0.016 (-0.125, 0.157) | 0.825 | 0.984 |
| C3 | 2.494 (-2.545, 7.533) | 0.332 | 0.916 |
| Log IL-6 | 0.056 (-0.089, 0.201) | 0.448 | 0.963 |
| Log TNF-α | -0.011 (-0.084, 0.062) | 0.769 | 0.984 |
| Log leptin | -0.029 (-0.178, 0.120) | 0.702 | 0.984 |
| Log adiponectin | -0.087 (-0.203, 0.030) | 0.146 | 0.75 |
| Log LAR | 0.057 (-0.134, 0.249) | 0.556 | 0.976 |
| Log resistin | 0.076 (-0.017, 0.170) | 0.11 | 0.673 |
| GlycA | 8.675 (-3.938, 21.287) | 0.177 | 0.784 |
| Log WBC | 0.067 (0.012, 0.122) | **0.016** | 0.168 |
| Log monocytes | 0.096 (0.030, 0.162) | **0.005** | 0.054 |
| Log basophils | 0.109 (-0.011, 0.229) | 0.075 | 0.558 |
| Log eosinophils | 0.120 (-0.008, 0.249) | 0.066 | 0.542 |
| Log neutrophils | 0.040 (-0.032, 0.111) | 0.277 | 0.908 |
| Log lymphocytes | 0.099 (0.033, 0.166) | **0.003** | **0.048** |
| Log NLR | -0.060 (-0.144, 0.025) | 0.166 | 0.784 |
| PAI-1 | 0.561 (-2.020, 3.142) | 0.67 | 0.984 |
| ***Neglect exposure*** | **β (95% CI)** | ***p*** | ***p* (FDR)** |
| Log CRP | 0.006 (-0.171, 0.182) | 0.95 | 1.0 |
| C3 | 5.616 (-0.695, 11.927) | 0.081 | 0.679 |
| Log IL-6 | 0.118 (-0.064, 0.300) | 0.203 | 0.922 |
| Log TNF-α | -0.012 (-0.104, 0.079) | 0.791 | 1.0 |
| Log leptin | -0.037 (-0.224, 0.151) | 0.701 | 1.0 |
| Log adiponectin | -0.015 (-0.162, 0.132) | 0.842 | 1.0 |
| Log LAR | -0.022 (-0.262, 0.219) | 0.859 | 1.0 |
| Log resistin | -0.046 (-0.164, 0.071) | 0.438 | 0.99 |
| GlycA | 10.703 (-5.163, 26.659) | 0.186 | 0.905 |
| Log WBC | 0.002 (-0.067, 0.070) | 0.959 | 1.0 |
| Log monocytes | 0.068 (-0.015, 0.151) | 0.111 | 0.749 |
| Log basophils | 0.048 (-0.102, 0.198) | 0.53 | 0.997 |
| Log eosinophils | 0.099 (-0.062, 0.260) | 0.227 | 0.922 |
| Log neutrophils | -0.009 (-0.098, 0.080) | 0.84 | 1.0 |
| Log lymphocytes | -0.014 (-0.097, 0.069) | 0.734 | 1.0 |
| Log NLR | 0.005 (-0.100, 0.110) | 0.923 | 1.0 |
| PAI-1 | -1.143 (-4.379, 2.093) | 0.488 | 0.993 |
| ***Household dysfunction exposure*** | **β (95% CI)** | ***p*** | ***p* (FDR)** |
| Log CRP | 0.045 (-0.086, 0.177) | 0.5 | 0.9221 |
| C3 | -0.195 (-4.898, 4.508) | 0.935 | 0.985 |
| Log IL-6 | 0.139 (0.004, 0.274) | **0.043** | 0.434 |
| Log TNF-α | -0.004 (-0.072, 0.064) | 0.902 | 0.985 |
| Log leptin | -0.140 (-0.279, -0.001) | **0.048** | 0.434 |
| Log adiponectin | -0.071 (-0.180, 0.038) | 0.204 | 0.863 |
| Log LAR | -0.070 (-0.248, 0.109) | 0.443 | 0.922 |
| Log resistin | 0.055 (-0.033, 0.142) | 0.218 | 0.863 |
| GlycA | 7.149 (-4.602, 18.901) | 0.233 | 0.863 |
| Log WBC | 0.041 (-0.010, 0.092) | 0.115 | 0.669 |
| Log monocytes | 0.067 (0.006, 0.129) | **0.032** | 0.394 |
| Log basophils | 0.117 (0.006, 0.228) | **0.039** | 0.434 |
| Log eosinophils | 0.168 (0.050, 0.287) | **0.006** | 0.118 |
| Log neutrophils | 0.054 (-0.012, 0.120) | 0.108 | 0.653 |
| Log lymphocytes | -0.027 (-0.089, 0.035) | 0.388 | 0.922 |
| Log NLR | 0.081 (0.003, 0.159) | **0.041** | 0.434 |
| PAI-1 | -1.216 (-3.622, 1.190) | 0.321 | 0.891 |
| Model 4: adjusted for age, education, anti-inflammatory medication use, type 2 diabetes, cardiovascular disease, cancer, smoking status, alcohol use, diet quality and BMI. Unstandardised β coefficients and 95% confidence intervals (CI) are shown.  Significant *p* in **bold**. | | | |
